# Hinokiflavone Inhibits Growth of Esophageal Squamous Cancer By Inducing Apoptosis *via* Regulation of the PI3K/AKT/mTOR Signaling Pathway

**DOI:** 10.3389/fonc.2022.833719

**Published:** 2022-02-01

**Authors:** Jida Guo, Shengqiang Zhang, Jun Wang, Pengfei Zhang, Tong Lu, Linyou Zhang

**Affiliations:** Department of Thoracic Surgery, The Second Affiliated Hospital of Harbin Medical University, Harbin Medical University, Harbin, China

**Keywords:** esophageal cancer, hinokiflavone, apoptosis, KEGG analysis, molecular docking, PI3K/AKT/mTOR signal pathway

## Abstract

**Background:**

Globally, esophageal cancer ranks as the seventh most common cancer. Esophageal squamous cell carcinoma (ESCC) is one of its major histological types. ESCC accounts for the vast majority of cases in China, and the mortality rate is high. Cisplatin, the standard adjuvant chemotherapy drug for ESCC, has a modest response rate due to the development of drug resistance. Hinokiflavone (HF) is a natural biflavonoid compound with anti-melanoma activity. However, its anti-tumor effect on ESCC and the underlying mechanisms remain largely unknown.

**Methods:**

The ESCC cell lines KYSE150 and TE14 were used. The cell counting kit-8 assay and flow cytometry analysis, along with colony formation, EdU, wound healing, and Transwell migration assays, were performed to assess cell characteristics (viability, migration, invasion, and apoptosis) following treatment with HF. Gene Ontology (GO), Kyoto Encyclopedia of Genes and Genomes (KEGG), western blotting, and molecular docking were used to investigate the pathways potentially modulated by HF. *In vivo* anti-tumor effects of HF were also investigated using a mouse xenograft model.

**Results:**

Our findings revealed that HF inhibited ESCC cell proliferation. Hoechst 33342 staining, annexin V-FITC/PI staining, and western blotting confirmed that HF causes caspase-dependent apoptosis. KEGG pathway enrichment analysis and western blotting indicated that the PI3K/AKT/mTOR pathway played an important role in the process of HF-induced apoptosis. Furthermore, HF effectively impaired the migration and invasion abilities of KYSE150 cells and downregulated the expression of the matrix metalloproteinases (MMP) MMP2 and MMP9. HF inhibited tumor growth and exhibited minimal toxicity in the organs of the KYSE150 xenograft model.

**Conclusion:**

This is the first study to demonstrate the inhibition of ESCC growth and progression by HF. The underlying mechanism is through blocking the PI3K/AKT/mTOR signaling pathway, thereby inhibiting cell proliferation and inducing apoptosis. HF can be used as a complementary/alternative agent for ESCC therapy.

## Introduction

Esophageal cancer is the sixth leading cause of cancer-related deaths and the seventh most common cancer worldwide (1). It is well known that there are two dominant histological types of esophageal cancer: esophageal adenocarcinoma and esophageal squamous cell carcinoma (ESCC). ESCC accounts for 90% of all esophageal cancer cases in Asia and Africa (1). In recent years, despite the development of multimodal treatment including surgery combined with chemoradiotherapy and targeted therapy, the prognosis of esophageal cancer patients remains poor due to the strong malignancy of this cancer (2, 3). Therefore, the development of novel therapeutic approaches for this disease is urgent and necessary.

**Figure 1:**
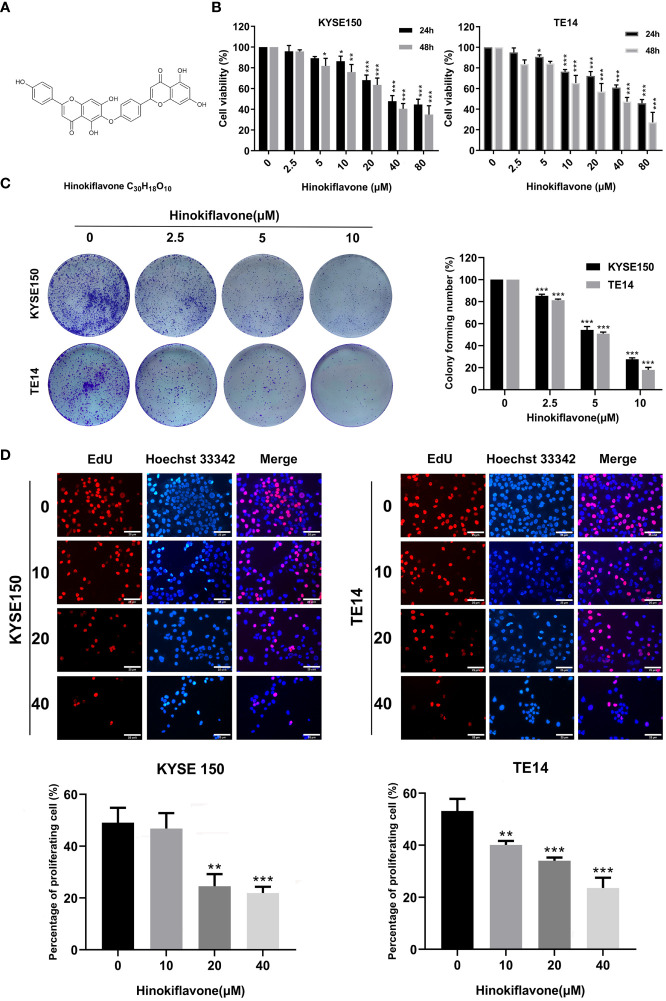
Effect of hinokiflavone (HF) on the proliferation of esophageal squamous cell carcinoma (ESCC) cells. **(A)** Two-dimensional chemical structure of HF (C_30_H_18_O_10_). **(B)** KYSE150 and TE14 cell lines were incubated with different concentrations of HF for 24 and 48 h Cell viability was evaluated using the CCK-8 assay. **(C)** HF reduced colony formation of ESCC cells. KYSE150 and TE14 cells were treated with 0, 2.5, 5, and 10 μM of HF and cultured for 9 days to form colonies. Colonies were stained with crystal violet and counted. **(D)** EdU assay analyzed the antiproliferative effect of HF on ESCC cells. Hoechst 33342 cell nuclei dye presented as blue fluorescence cells represent total cells, and EdU positive cells presented as red fluorescence represent proliferating cells. Magnification, ×200; scale bars = 25 µm. All the above experimental data are presented as the mean ± SD of three independent experiments. *P < 0.05; **P < 0.01; ***P < 0.001 compared to vehicle control (0 μM) group.

Natural products have a long history of use for the treatment of human disease, which is of great value for drug discovery and development (4). Moreover, many common chemotherapeutic compounds have been derived from natural products (5, 6). Hinokiflavone (HF) (**Figure 1A**, C_30_H_18_O_10_) has been extracted from several plants, including *Selaginella tamariscina, Juniperus phoenicea*, and *Rhus succedanea*, with high stability. It exhibits several biological activities including cytotoxicity (7), anti-HIV-1 reverse transcriptase activity (8), and antioxidant activity (9). Sui et al. (10) reported that the ethyl acetate extract of *Selaginella doederleinii* Hieron inhibits proliferation of A549 cell lines by inducing apoptosis. Magne et al. (11) also identified natural flavonoids in HF that are useful therapeutic agents for breast cancer. In addition, HF might also be a novel compound to treat melanoma by cell cycle arrest, inducing apoptosis and blocking cell migration and invasion (12). However, the antitumor effect of HF in ESCC and its specific targeted signaling pathways have not been investigated.

Multiple studies have demonstrated that phosphoinositides, the multiphosphorylated derivatives of phosphatidylinositol, are membrane-bound signaling molecules that play a crucial role in a variety of cellular biological processes (13–15). Under general physiological conditions, phosphatidylinositol-3-kinase (PI3K) activates AKT (protein kinase B) by phosphorylating Thr 308 and Ser 473 (p-AKT) (16, 17). p-Akt then phosphorylates several of its downstream effector proteins, such as tuberous sclerosis complex 2 (TSC2) and glycogen synthase kinase 3 (GSK3α/β), and these activated proteins subsequently regulate cell growth, survival, and apoptosis (18, 19). Whereas mammalian target of rapamycin (mTOR) was shown to be the major signaling molecule downstream of TSC2, targeting regulation of the PI3K/Akt/mTOR pathway has been proven effective in cancer therapies (20, 21). Accumulating evidence has also shown that the PI3K/Akt signaling pathway is activated in multiple tumor types and contributes to tumor progression by promoting tumor cell proliferation, apoptosis resistance, and distant metastasis (22, 23). Moreover, the PI3K/AKT signaling pathway plays a vital role in ESCC growth and metastasis (24, 25).

In the current study, we extensively explored the anti-tumor effects of HF on human esophageal cancer cells *in vitro* and *in vivo* as well as the underlying molecular mechanisms. We found that HF exhibited a strong growth inhibitory effect on two ESCC cell lines (KYSE150 and TE14) and the PI3K/AKT/mTOR signaling pathway plays a crucial role in HF-induced apoptosis in these cells. These results indicate that HF has potential anti-tumor activity, which can be exploited to develop effective therapeutic strategies for ESCC.

## Materials and Methods

### Reagents, Antibodies and Kits

HF of 98% purity was purchased from Chengdu Biopurify Phytochemicals, Ltd. (Chengdu, China). Dimethylsulfoxide (DMSO), RPMI 1640 medium, and fetal bovine serum (FBS) were purchased from Sigma Chemical Co. (St Louis, MO, USA). Antibodies against β-actin, phosphorylated PI3K [p-PI3K (Tyr458 and Tyr199)], PI3K, phosphorylated AKT [p-AKT (Ser473 and Thr308)], AKT, cleaved Caspase 3 (c-Caspase 3), phosphorylated mTOR [p-mTOR (Ser2448)], mTOR, matrix metalloproteinase-2 (MMP2), and MMP9 were purchased from Cell Signaling Technology, Inc. (Danvers, MA, USA). Antibodies against Bcl-2 and Bax were purchased from Proteintech Group, Inc. (Chicago, IL, USA). RIPA lysis buffer, phenylmethanesulfonyl fluoride (PMSF), and the bicinchoninic acid (BCA) protein assay kit were from Beyotime (Nanjing, China). Enhanced chemiluminescence detection reagent was obtained from GE Healthcare Life Sciences Inc. (Marlborough, MA, USA). SC-79 (AKT activator) was purchased from MedChemExpress (Shanghai, China). Cell Counting Kit-8 (CCK-8) was obtained from Dojindo Laboratories (Kumamoto, Japan). Hoechst33342 and BeyoClick™ EdU Cell Proliferation Kit with Alexa Fluor 594 and the Annexin V-fluorescein isothiocyanate (FITC)/propidium iodide (PI) apoptosis detection kit were purchased from Beyotime Biotechnology (Jiangsu, China).

A stock solution of HF dissolved in DMSO (40 mM) was stored at -20°C and diluted with medium for experimental applications.

### Cell Culture

KYSE150 and TE14 cell lines were purchased from the Cell Bank of the Chinese Academy of Sciences. Cells were cultured in RPMI 1640 medium with 10% FBS and 1% penicillin-streptomycin at 37°C in a humidified incubator with 5% CO_2_ and 95% humidity.

### Cell Proliferation Assay

Cell viability was determined using the CCK-8 assay. KYSE150 and TE14 cells were seeded at a density of 1×10^4^ cells/well in 96-well plates. After 24 h of incubation, the cells were treated with 0, 2.5, 5, 10, 20, 40, and 80 μM of HF. Controls were treated with RPMI medium containing DMSO only. After 24 h and 48 h, old medium was aspirated and 100 μL of CCK-8 working solution, diluted to 10% in culture medium, was added to each well and further incubated for 4 h at 37°C in a humidified incubator. The absorbance at 450 nm was measured using a microplate reader. IC_50_ values were calculated with Graphpad Prism 8 using data obtained from three independent experiments.

### Colony Formation Assay

KYSE150 and TE14 cells were plated in 6-well plates (1.0×10^3^ cells/well). After 24 h of incubation, the cells were incubated with various concentrations (0, 2.5, 5, and 10 μM) of HF for 9 days. Cells were fixed with 4% paraformaldehyde and then stained with 0.5% crystal violet for 15 min, after which a dissecting microscope was used to count colonies (> 50 cells).

### EdU Assay

The EdU assay was performed to analyze proliferating cells by evaluating the incorporation of fluorescent labeled EdU into replicating DNA in the S phase of cell cycle. The BeyoClick™ EdU Cell Proliferation Kit with Alexa Fluor 594 was used for this purpose, according to the manufacturer’s instructions. The maximum excitation wavelength of Alexa 594 is 590 nm, and the maximum emission wavelength is 615 nm. Briefly, KYSE150 and TE14 cells were seeded in 96-well plates at a cell density of 5.0×10^3^ cells/well and treated with different concentrations of HF for 24 h. Edu was added to the culture medium and brought to a final concentration of 10 μM, and the cells were cultured for an additional 2.5 h at 37°C. Afterwards, cells were washed twice using phosphate-buffered saline (PBS) and fixed by 4% paraformaldehyde for 15 min at approximately 25°C. After co-staining with Hoechst 33342, the cells were imaged using a fluorescence microscope (Leica, Wetzlar, Germany).

### Morphological Analysis by Hoechst 33342 Staining

Alterations in cell morphology, including cell shrinkage and apoptotic body formation, were analyzed after Hoechst 33342 staining to identify apoptotic cells. KYSE150 and TE14 cells were seeded in 96-well plates at a density of 5.0×10^3^ cells/well and cultured for 24 h. After treatment with different concentrations of HF (0, 10, 20, and 40 μM) for 24 h, the cells were washed twice using PBS and fixed in 4% paraformaldehyde for 15 min. Then, the cells were stained using Hoechst 33342 working solution diluted well according to the instructions for 15 min at 25°C and analyzed under a fluorescence microscope (Zeiss, Axiovert 200, Oberkochen, Germany).

### Quantification of Apoptosis

Further verification of HF-induced apoptosis was carried out using the Annexin V-FITC/PI apoptosis detection kit. After treatment with HF (0, 10, 20, and 40 μM) for 24 h, KYSE150 and TE14 cells at a cell density of 1.0×10^6^ cells/well were trypsinized, collected by centrifugation, and washed twice with ice-cold PBS. The cells were then co-stained with 5 μL FITC-Annexin V and 5 μL PI for 15 min at 20–25 °C in the dark. The apoptosis rate was measured by flow cytometry (CytoFLEX, Beckman Coulter, Brea, CA, USA).

### Gene Ontology and Kyoto Encyclopedia of Genes and Genomes Pathway Enrichment Analyses

A total of 355 and 100 potential targets for HF were searched from the Pharma Mapper (http://www.lilab-ecust.cn/pharmmapper/) and Swiss target prediction (http://www.swisstargetprediction.ch/) databases, respectively. GO and KEGG pathway enrichment analyses was performed using the DAVID database (26, 27).

### Molecular Docking

The 3D structures of PI3K (code 6pys) and AKT1 (code 4ejn) were downloaded from the Protein Data Bank (https://www.rcsb.org/). Chemical structure data of HF was obtained from PubChem (http://pubchem.ncbi.nlm.nih.gov/). Molecular docking was implemented using the LibDock module of the Discovery Studio 2016, and the results were evaluated using the LibDock score. A higher libdock score indicates a higher activity of the small molecule (such as HF) binding to the target protein.

### Western Blot Analysis

KYSE150 and TE14 cells at a cell density of 5.0×10^6^ cells/well were treated with various concentrations of HF (0, 10, 20, and 40 μM) for 24 h. Cells were washed twice using PBS and lysed by adding RIPA cell lysis buffer containing PMSF. The cell lysate was centrifuged at 12,000 × *g* for 20 min at 4°C, after which the supernatant was gently aspirated using a pipette. The total protein content in the supernatant was measured using the BCA protein assay kit. Equal amounts of protein (20 μg) were separated by SDS-PAGE (8%–12%). The separated proteins were then transferred onto a PVDF membrane. The membranes were blocked with Quickblock blocking buffer (Beyotime Biotechnology, China) for 0.5 h and incubated with specific primary antibodies overnight at 4°C on a shaker. Membranes were washed three times for 10 min each with Tris-buffered saline-Tween 20 (TBST), completed by incubation with horseradish peroxidase (HRP)-conjugated secondary antibody for 1 h at approximately 25°C. Our study used the following primary antibodies: β-actin (1:1,000), phosphorylated PI3K (p-PI3K) (Tyr458, 1:1,000 and Tyr199, 1:1,000), PI3K (1:1,000), phosphorylated AKT (p-AKT) (Ser473, 1:1,000; Thr308, 1:1,000), AKT (1:1,000), c-Caspase 3 (1:1,000), p-mTOR (Ser2448, 1:1,000), mTOR (1:1,000), matrix metalloproteinase-2 (MMP2) (1:1,000), MMP9 (1:1,000), Bcl-2 (1:2,000), and Bax (1:1,000). The secondary antibody was HRP-conjugated goat anti-rabbit antibody (1:10,000; Cell Signaling Technology, Inc.). After three 10 min washes with TBST, the protein band was visualized using enhanced chemiluminescence detection reagent (GE Healthcare Life Sciences). Finally, protein bands were quantified by densitometric analysis using ImageJ software, and the protein amounts were expressed relative to the corresponding reference protein.

### Wound-Healing Migration Assay

KYSE150 cells were grown to a density of approximately 80-90% in 6-well plates, after which the cell monolayer was scraped using a sterile 100-μL pipette tip to create “wounds”. Reduced serum medium (1 mL) containing HF (0, 10, 20, or 40 μM) was gently added to the wells along the lateral wall to ensure that it covered the entire bottom. After incubation for 24 h in an incubator, the cells were washed and fixed with 4% paraformaldehyde (1 mL/well), and images were acquired under a microscope (Zeiss, Jena, Germany). Cell movement distances were measured by ImageJ (v1.8.0).

### Cell Migration and Invasion Assay

Transwell migration and invasion assays were implemented by using Boyden chamber (8 μm pore size, Corning, NY, USA). For migration assay, KYSE150 cells (2.0 ×10^4^ cells in 100 μL serum-free medium) were added into the upper chamber, and 600 μL of medium with 10% FBS was added into the lower chamber. For invasion assay, the upper surface of the Transwell membrane was coated with Matrigel (BD Biosciences, Franklin lakes, NJ, USA) diluted 1:8 with serum-free medium before adding cells. For both assays, various concentrations of HF (0, 10, 20, and 40 μM) were added into the lower chamber. After incubation for 24 h, the medium was discarded, the cells were gently removed from the upper surface of the Transwell membrane with a cotton swab, and the migrated or invaded cells on the lower surface were fixed with 4% paraformaldehyde, after which they were stained with 0.5% crystal violet. Using microscopy, the number of stained cells on the lower surface was counted in five random fields per Transwell membrane (×100 magnification), and the Image J software was used to enumerate the migrated/invaded cells.

### Mouse Xenograft Model

All experimental procedures and manipulations involving animals were performed in compliance with the guidelines of the National Institutes of Health Guide for the Care and Use of Laboratory Animals. Moreover, all animal experiments were approved by the Institutional Animal Care and Use Committee of the Second Affiliated Hospital of Harbin Medical University (SYDW2021-046). Female BALB/c-nu athymic mice, at 6 weeks and weighing 14-17 g, were purchased from Beijing Vital River Laboratory Animal Technology Co., Ltd. (Beijing, China). Mice were maintained in a specific pathogen-free (SPF) grade rearing environment using sterile water and food feeding. KYSE150 cells (1.0×10^7^ cells in 100 µL PBS) were injected subcutaneously into the right shoulders of the mice (n=18). Tumor size was measured every 3 days using a caliper. The formula for calculating tumor volume was as follows: V = 0.5×L×W^2^ (L, tumor longest diameter; W, tumor shortest diameter). When the tumor volume reached 50 mm^3^, mice were randomized into three groups (n=6/group; control, 25 mg/kg HF, and 50 mg/kg HF). Next, HF (25 mg/kg and 50 mg/kg in 200 μL saline) was administered by intraperitoneal injection to the two treatment group mice daily for 21 days. The control mice received saline alone. The body weight and tumor volume of mice were measured every 3 days. After completion of drug administration, all animals were fed normal diet for another 3 days, after which they were euthanized by cervical dislocation. The tumors were excised, weighed, and immunohistochemical staining was performed. The internal organs were collected for hematoxylin and eosin (H&E) staining.

### Immunohistochemistry

Tumor tissues from mice were formalin fixed, then embedded in paraffin and sectioned at 4 µm thin sections were used for examination. For staining, tissue sections were deparaffinized and rehydrated following standard protocols. Antigen retrieval of tissues in our study was performed by boiling in sodium citrate buffer (10 mM, pH 6.0) for 10 min, after which the samples were treated with 3% H_2_O_2_ for an additional 10 min. Each section was blocked with 10% goat serum albumin in a humidified chamber for 1 h at approximately 20–25°C. Then, tissue sections were incubated with specific primary antibodies (p-AKT, p-mTOR, Bax, and c-Caspase 3) overnight at 4°C in a humidified chamber. After further washing, the sections were incubated with secondary antibody for 30 min at approximately 25°C, followed by incubation with 3,3’-diaminobenzidine and counterstaining with hematoxylin. Stained sections were imaged under a microscope (Leica, DM4000B).

### H&E Staining

Mouse organs (heart, liver, spleen, lung, and kidney) were fixed in formaldehyde, embedded in paraffin, and sectioned at 4 μm thickness. After deparaffinization and rehydration, tissue sections were stained with hematoxylin for 10 min, 1% ethanol-hydrochloric acid for 30 s, and eosin solution for 3 min. The sections were dehydrated in graded alcohol, after which they were cleared with xylene and finally mounted using neutral balsam.

### Statistical Analysis

All data are presented using the mean ± SD of three independent experiments. Statistical analysis was performed using GraphPad Prism version 8 (GraphPad Software, San Diego, CA, USA). Student’s *t*-test was used to compare two groups, and one-way analysis of variance was used for multiple comparisons. Statistically significant *P-*values are labeled as follows: *P < 0.05, **P < 0.01, ***P < 0.001.

## Results

### HF Inhibits the Proliferation of ESCC Cells

The chemical structure of HF was drawn with ChemDraw software as shown in **Figure 1A**. KYSE150 and TE14 cell lines were treated with HF at different concentrations (0, 2.5, 5, 10, 20, 40, or 80 μM) for 24 or 48 h, and cell proliferation was assessed by CCK-8 assay, as described in the Methods. As shown in **Figure 1B**, HF significantly inhibited the cell viability of both ESCC cell lines in a concentration- and time-dependent manner. Furthermore, the IC_50_ values for KYSE150 cells treated with HF for 24 h and 48 h were 27.92 μM and 24.91 μM, respectively, while the respective values for TE14 cells were 26.21 μM and 22.07 μM. Long-term cell viability assays (colony formation assays) showed that KYSE150 and TE14 cells formed significantly fewer colonies with increasing HF concentrations. (**Figure 1C**). The anti-proliferative activity of HF was further verified using an EdU assay. As shown in **Figure 1D**, the proportion of proliferating KYSE150 and TE14 cells decreased from 49.1% and 53.2% to 21.9% and 23.6%, respectively, after 40 μM HF treatment for 24 h. Taken together, these results indicate that HF effectively inhibits ESCC cell proliferation.

### HF Induces Apoptosis in ESCC Cells

**Figure 2:**
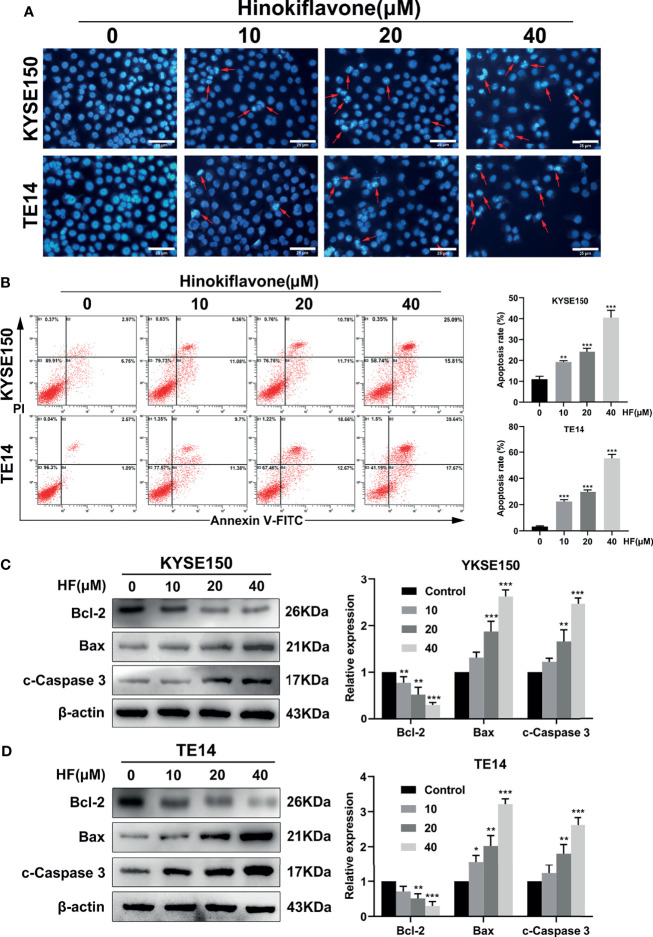
Induction of apoptosis in ESCC cells by HF. **(A)** Hoechst 33342 nuclear dye was used to stain the KYSE150 and TE14 cells and utilized fluorescence microscopy to detect HF induced apoptotic nuclear morphological alterations (indicated by red arrows). Magnification, ×200; scale bars = 25 µm. **(B)** Percentage of KYSE150 and TE14 cells apoptosis after HF treatment was measured using Annexin V-fluorescein isothiocyanate (FITC)/propidium iodide (PI) double staining and analyzed by flow cytometry. **(C, D)** Expression levels of Bcl-2, Bax, and c-Caspase 3 were detected by western blot and quantitated *via* densitometric analysis using ImageJ software, while the expression level of β-actin was used as an internal control. The relative expression of experimental histones was calculated according to the protein scale set to 1 for 0 μM control group. All the above experimental data are presented as the mean ± SD of three independent experiments. *P < 0.05; **P < 0.01; ***P < 0.001 compared to vehicle (0 μM) group.

We next explored whether HF induces apoptosis in ESCC cells. Induction of apoptosis by HF was first evaluated by Hoechst 33342 staining assay. As shown in **Figure 2A**, HF-treated KYSE150 and TE14 cell cultures contained dense and heavily stained apoptotic cells, suggesting that HF might cause various degrees of cell shrinkage, nuclear fragmentation, and condensed nuclei formation. Furthermore, flow cytometric analysis of Annexin V-FITC and PI staining revealed that the ratio of early and late apoptotic cells increased in a dose-dependent manner after treatment with HF for 24 h. As shown in **Figure 2B**, the mean apoptosis ratios (from three independent experiments) were 11.0% (0 μM), 19.2% (10 μM), 24.2% (20 μM), 40.6% (40 μM) for KYSE150 cells; 3.3% (0 μM), 22.3% (10 μM), 29.9% (20 μM), and 55.6% (40 μM group) for TE14 cells.

Next, apoptosis-related proteins Bcl-2 (anti-apoptotic) and Bax (pro-apoptotic) were analyzed by western blot. Caspase-3, which is a member of the caspase family and is cleaved during apoptosis (28–30), was also analyzed by western blot. Treatment of KYSE150 and TE14 cells with HF significantly reduced the protein level of Bcl-2, while increasing the protein levels of Bax and c-Caspase 3 in a dose-dependent manner (**Figures 2C, D** **)**. These results indicate that HF induced apoptosis of ESCC cells involves the mitochondrial pathway.

### GO and KEGG Pathway Enrichment Analyses

**Figure 3:**
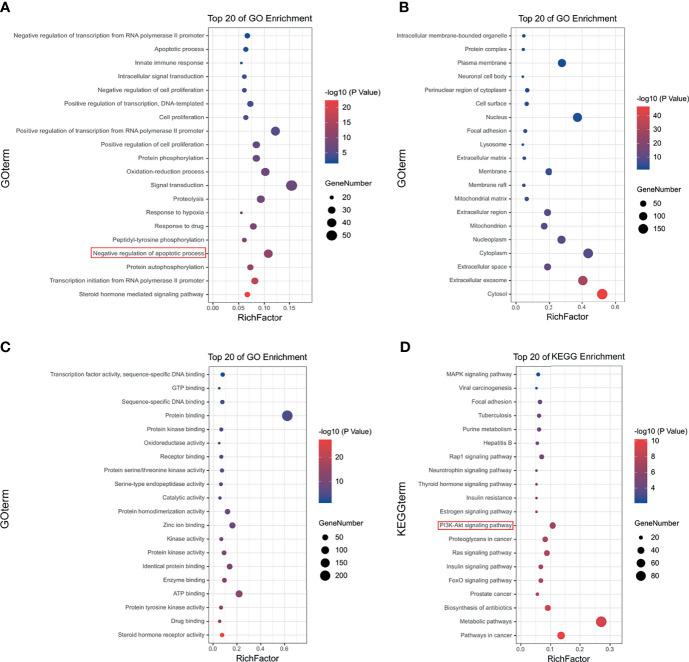
Gene Ontology (GO) and Kyoto Encyclopedia of Genes and Genomes (KEGG) pathway analyses of HF. Top 20 signaling pathways of HF in **(A)** GO-BP pathway analysis, **(B)** GO-CC pathway analysis, and **(C)** GO-MF pathway analysis. **(D)** Top 20 signaling pathways of HF in KEGG enrichment pathway analysis.

GO enrichment analysis of HF was performed on the 355 putative targets of HF that were collected from the Pharma Mapper database to determine the relative significance with regards to biological processes, cell components, and molecular functions. The top 20 significantly enriched GO terms are listed in **Figures 3A–C** and **Table S1**. KEGG pathway enrichment analysis of HF was performed, and the top 20 targets are listed in **Figure 3D** and **Table S2**. The results show pathway enrichment for the GO and KEGG terms “negative regulation of apoptotic process,” “pathways in cancer,” and “PI3K-AKT signaling pathway,” based on the Pharma Mapper database. Similarly, we obtained 100 putative targets of HF from the Swiss database. The GO enrichment analysis of the Swiss database is shown in **Figures S1A–C** and **Table S3**, and the KEGG pathway enrichment analysis is shown in **Figure S1D** and **Table S4**. Interestingly, the results analyzed according to the Swiss database were similar to those analyzed by the Pharma Mapper database. In summary, the analysis results of both databases indicate that HF might target the PI3K-AKT signaling pathway, leading to induction of apoptosis.

### HF Is Predicted to Have Direct Interaction With PI3K and AKT1

**Figure 4:**
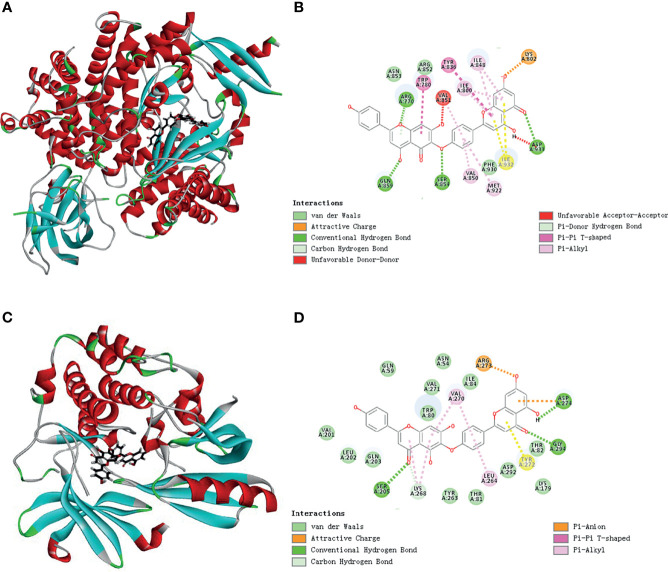
Schematic representation of three- and two-dimensional molecular docking models. **(A)** Three-dimensional view of HF and PI3K docking scenario. **(B)** Two-dimensional view of HF and PI3K docking scenario. **(C)** Three-dimensional view of HF and AKT1 docking scenario. **(D)** Two-dimensional view of HF and AKT1 docking scenario.

To predict the possible mutual binding mode of HF with PI3K and Akt, we further used molecular docking analysis. As shown in **Figure 4A**, HF was predicted to have a strong interaction with PI3K (LibDock score 115.982). The molecular docking of other conformations of HF with the protein PI3K is shown in **Figures S2A, B**. The binding affinity was attributed to the following: hydrogen bonding with the ARG-770, GLN-895, SER-854, and ASP-933 residues; the pi-pi T-shaped interaction with the TRP-780 and TYR-836 residues; the pi-alkyl interactions with the ILE-800, ILE-848, VAL-850, and MET-922 residues of PI3K (**Figure 4B**). Moreover, the analysis results show that HF could also interact strongly with protein AKT1 (LibDock score 143.752, **Figure 4C**). Molecular docking of other conformations of HF with AKT1 is shown in **Figures S2C, D**. The binding affinity was attributed to the following: hydrogen bonding with the SER-205, GLY-294, and ASP-274 residues; the pi-alkyl interactions with the LYS-268, VAL-270, and LEU-264 residues, and pi-anion interactions with the ARG-273 and ASP-274 residues of AKT1 (**Figure 4D**). Therefore, in common with the results of KEGG pathway enrichment analysis, HF showed high binding activity with PI3K and AKT in this study.

### HF Inhibits the PI3K/AKT/mTOR Pathway in ESCC Cells

**Figure 5:**
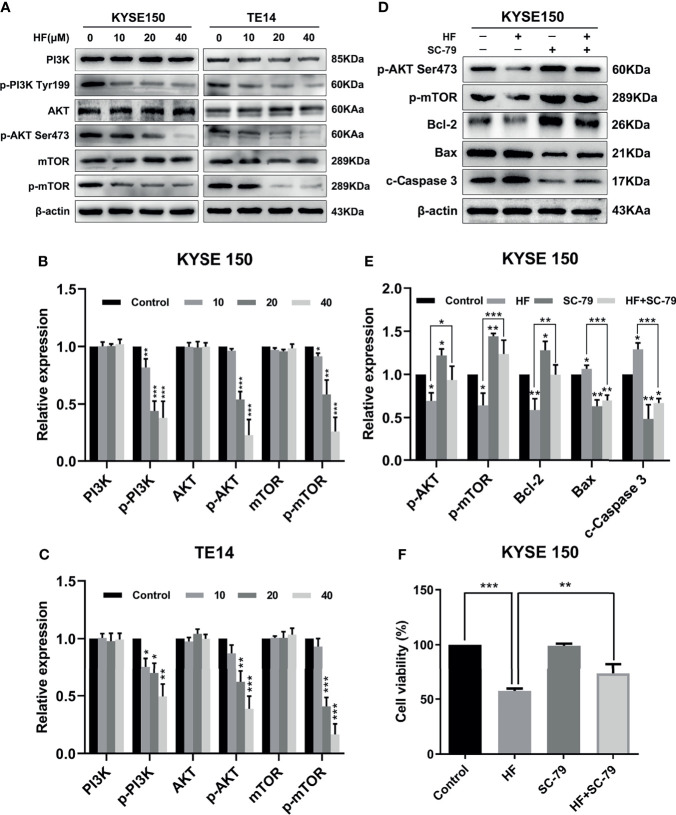
HF induces apoptosis of ESCC cells by negatively regulating the PI3K/Akt/mTOR pathway. **(A–C)** KYSE150 and TE14 cells received various concentrations of HF intervention for 24 h, and the expression of pathway related proteins PI3K, p-PI3k, Akt, p-Akt, mTOR, and p-mTOR was detected by western blotting and quantified *via* densitometric analysis. **(D, E)** KYSE150 cells were pretreated with SC-79 (10 μM) for 24 h, and the expression of apoptosis related proteins Bcl-2, Bax and c-caspase 3 was determined by western blotting. **(F)** KYSE150 cells were first cultured with HF with or without addition of SC-79 (10 μM) for 24 h, and cell viability was detected using CCK-8 assays. The above experimental data are presented as the mean ± SD of three independent experiments. *P < 0.05; **P < 0.01; ***P < 0.001 compared to the control group.

To verify the interaction of HF with PI3K/AKT, we analyzed HF-treated KYSE150 and TE14 cells *via* western blotting. Our results showed that the protein levels of p-PI3K, p-AKT, and p-mTOR in KYSE150 and TE14 cells were significantly reduced by HF treatment at 10, 20, and 40 μM, whereas total expression levels of PI3K, AKT, and mTOR were not significantly changed (**Figures 5A–C**). To verify whether HF induces apoptosis through the inhibition of PI3K/AKT/mTOR pathway, KYSE150 cells were treated with HF in the presence or absence of SC-79, a specific AKT activator (31). As shown in **Figures 5D, E**, SC-79 effectively enhanced the levels of phosphorylated proteins including p-AKT and p-mTOR. Meanwhile, pretreatment with SC-79 improved the inhibitory effect of HF on the protein expression of p-AKT, p-mTOR, and Bcl-2. Furthermore, the effect of HF to increase the expression amounts of apoptosis-related proteins (Bax and c-Caspase 3) could be reversed by pretreatment with SC-79 as well. We also found an increased viability of KYSE150 cells in the HF and SC-79 co-treatment group compared with HF alone treatment (**Figure 5F**), suggesting that activation of AKT attenuated the ability of HF to inhibit cell proliferation. These results indicate that the anticancer effect of HF on ESCC involves regulation of PI3K/AKT/mTOR pathway.

### HF Suppresses Migration and Invasion in ESCC Cells

**Figure 6:**
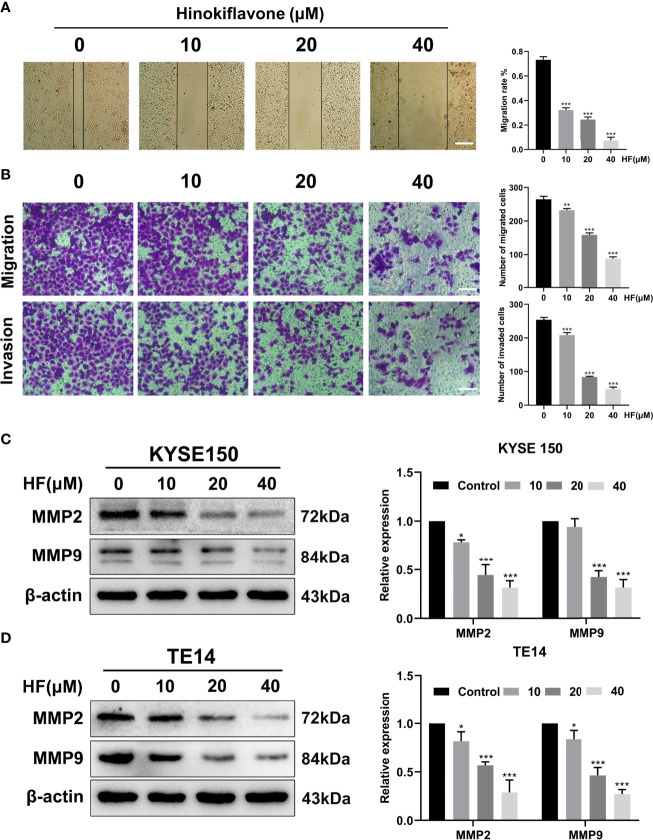
HF inhibits ESCC cell migration and invasion. **(A)** Cell migration was measured in a scratch-wound assay. KYSE150 cells were cultured until reaching approximately 80-90% cell density, the culture was scratched as described in Methods, and further cultured with various concentrations of HF (0, 10, 20, and 40 μM) for 24 h, after which the cells were fixed and photographed. Magnification, ×100; scale bars = 100 µm. The distance of cell migration from the same region was quantified using ImageJ. **(B)** KYSE150 cells were placed in the top chamber on the Transwell membrane with serum-free medium and the upper surface of the Transwell membrane was coated with/without Matrigel. Cells were treated using HF with a concentration gradient for 24 h, and then the cells were fixed and photographed using a microscope after crystal violet staining. Magnification, ×100; scale bars = 100 µm. Migrated and invaded cells were counted as described in the Methods. **(C, D)** KYSE150 cells and TE14 cells were treated with various concentrations of HF for 24 h, and then the cells were collected for protein extraction. The extracted proteins were later used for western blot analysis to determine the protein expression levels of MMP2 and MMP9. All the above experimental data are presented as the mean ± SD of three independent experiments. *P < 0.05; ***P < 0.001 compared to 0 μM vehicle group.

Esophageal cancer patients with distant metastases have a very poor prognosis (32), and the migration and invasion abilities of tumor cells are vital factors affecting the process of tumor metastasis (33). Therefore, we evaluated whether HF could inhibit the migration and invasion of ESCC cells. As revealed by wound-healing assay (**Figure 6A** **)**, the migratory ability of KYSE150 cells gradually became weaker with higher HF concentrations. Moreover, compared with the control group, HF treatment significantly inhibited the Transwell migration and invasion of ESCC cells in a dose-dependent manner (**Figure 6B**). Studies have shown that MMP family proteins play an important role in the regulation of cell migration and invasion abilities, and that MMPs are always upregulated in invasive epithelial cancers (34, 35). Therefore, we explored whether the protein levels of MMP2 and MMP9 are associated with alterations in ESCC cell migration and invasion following treatment with HF. As shown in **Figures 6C, D**, we found that HF significantly downregulated the expression of MMP2 and MMP9 in ESCC cells. Altogether, these results suggest that HF possesses an effective ability to inhibit ESCC cell migration and invasion.

### Antitumor Effect of HF in Mouse Xenograft Model of ESCC

**Figure 7:**
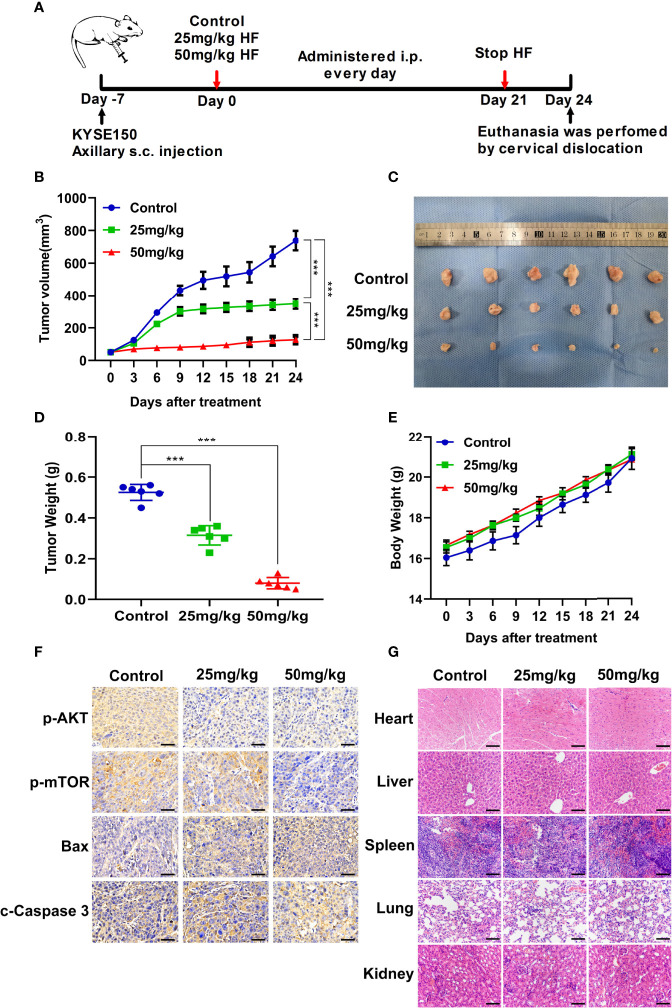
HF suppresses the growth of mouse ESCC xenograft tumors. **(A)** BALB/c-nu mice implanted with KYSE150 xenograft tumors were treated daily with an equal volume of saline (control group) or HF (25 or 50 mg/kg) by intraperitoneal injection for total 21 days. **(B)** The tumor volume was measured every 3 days, and the difference in tumor volume between HF treated and control mice is shown. ***P < 0.001. **(C)** After euthanasia of mice, subcutaneous xenografts were removed and photographed. **(D)** The removed xenograft tumors were weighed and graphed for statistical analysis. ***P < 0.001 vs the control group. **(E)** Body weights of tumor-bearing mice were measured every 3 days in the HF-treated and control groups. **(F)** Expression levels of p-AKT, p-mTOR, Bax, and cleaved-Caspase 3 were detected by immunohistochemistry in the xenograft tumors. Magnification, ×400; scale bars = 50 µm. **(G)** H&E staining of the heart, liver, spleen, lung, and kidney of experimental mice shows no pathological changes in the organ tissues of any group. Magnification, ×200; scale bars = 100 µm.

To investigate whether the antitumor effect of HF *in vivo* is consistent with its *in vitro* effects, ESCC xenografts were established by subcutaneously transplantation of KYSE150 cells into BALB/c-nu mice, followed by treatment with HF (saline control, 25 mg/kg HF, 50 mg/kg HF) for 3 weeks, as described in the Methods (**Figure 7A**). Results from the animal experiments showed that transplanted tumors in the HF intervention groups grew much slower than those in the control group (**Figures 7B–D**). The mean size (or weight) of the tumors in HF 25 mg/kg, and HF 50 mg/kg group was reduced to 348.9 mm^3^ (or 0.315 g) and 126.9 mm^3^ (or 0.08 g), compared with 738.9 mm^3^ (or 0.525 g) in the control group (**Figures 7B, D** **)**. As a crucial indicator of health, the average body weights of the control and HF-treated mice were not significantly different at all time points (**Figure 7E**). Moreover, immunohistochemical analysis showed that HF suppressed the protein expression levels of p-AKT and p-mTOR, while upregulating those of Bax and c-Caspase 3 (**Figure 7F**). As shown in **Figure 7G**, the results of H&E staining experiments on the heart, liver, spleen, lung, and kidney of mice exhibited no obvious pathological changes, such as necrosis, edema, or hemorrhage, indicating that HF possessed no major organ-related toxicity. Consistent with the experimental results *in vitro*, these data suggest that HF inhibits tumor growth by inducing apoptosis and does not have apparent adverse effects at the dose tested.

## Discussion

Despite progressive advances in the treatment of esophageal cancer, its mortality rate remains high worldwide. Currently, surgery and chemotherapy remain the main treatment modalities for esophageal cancer patients. However, since chemotherapeutics have their own disadvantages, such as drug resistance and systemic toxicity, more effective therapeutic strategies with fewer side-effects are urgently needed. Many studies have demonstrated the promising antitumor activity of some naturally active compounds (36). Our study is the first to indicate that HF inhibits the growth of ESCC cells. Furthermore, we comprehensively and profoundly explored the detailed molecular mechanisms underlying the antitumor effects of HF on ESCC *in vitro* and *in vivo* experiments.

In this study, the CCK-8 and colony formation assays demonstrated that HF had an effective anti-proliferative effect on KYSE150 and TE14 cells, which was validated by the EdU assay results. To investigate the anti-proliferation mechanism of HF in ESCC cells, we used Hoechst 33342 staining assay to identify apoptotic features of cell shrinkage, nuclear fragmentation, and condensed nuclei formation in HF-treated KYSE150 and TE14 cells. Flow cytometric analysis further illustrated induction of apoptosis in ESCC cells by HF in a concentration-dependent manner. Apoptosis is an orderly enzymatic cascade that induces DNA fragmentation into characteristic nucleosome fragments and culminates in cell death (30, 37, 38). Previous study has shown that apoptosis *via* the mitochondrial pathway is commonly regulated by Bcl-2 family proteins, with representative proteins such as the anti-apoptotic protein Bcl-2 and the pro-apoptotic protein Bax (39). In this context, our experimental results proved that HF intervention can cause a decreased expression of Bcl-2 protein and an increase in Bax and c-Caspase 3 expression, suggesting that HF induced apoptosis of ESCC cells occurs through the mitochondrial pathway.

We next used two databases, the Pharma Mapper and Swiss database, to screen the putative molecular targets of HF. The GO functional enrichment analysis showed that the predicted potential targets of HF were significantly enriched in “negative regulation of apoptotic process.” Furthermore, KEGG pathway analysis revealed that HF might affect the PI3K-AKT signaling pathway. Our molecular docking analysis further indicated that HF can dock with PI3K and AKT1. Western blot analysis demonstrated a progressive decrease in p-PI3K, p-AKT, and p-mTOR protein levels with increasing concentrations of HF in KYSE150 and TE14 cells (**Figure 5A**). Interestingly, western blot analysis showed that the activation (phosphorylation) of PI3K, as indicated by levels of p-PI3K, was inhibited by HF, whereas expression of PI3K protein itself was not significantly altered. This suggested that HF can regulate post-translational modification of PI3K by physically interacting with the protein, as shown by the results of the GO enrichment, KEGG pathway, and molecular docking analyses. It is plausible that HF may directly attenuate the phosphorylation of PI3K at tyrosine 199, as reported in another study (40). The PI3K/Akt/mTOR pathway is a crucial signaling cascade in living organisms that has to be activated in a variety of cancers and to regulate cell proliferation, invasion, and migration (20, 41). We observed (**Figures 5D–F** **)** that SC-79 usage could activate the PI3K/AKT/mTOR pathway and partially counteract the pro-apoptotic and anti-proliferative effects of HF in ESCC cells. Overall, our results clearly show that the PI3K/AKT/mTOR pathway was inhibited by HF, which induced apoptosis in ESCC cells.

Metastasis is known to be a multistep biological process wherein subsets of cancer cells spread from the primary tumor to distant tissues or other organs to form metastases affects the body’s normal physiological functions, which is the leading cause of cancer-related death (42). Moreover, for esophageal cancer patients, approximately 50% have already developed distant metastasis by the time of initial diagnosis (43, 44). Members of the MMP family of proteins, particularly MMP2 and MMP9, play a crucial role in cancer metastasis (34, 45, 46). The results of the migration and invasion assay we performed showed HF exhibited an obvious inhibitory effect on the metastatic potential of ESCC cells. We also found that HF significantly reduced MMP2 and MMP9 expression in KYSE150 and TE14 cells. These results suggest that HF can significantly hinder the metastasis of ESCC cells.

Finally, to verify the antitumor effect of HF *in vivo*, an esophageal cancer cell xenograft model was used in our study. Our experimental results showed that HF treatment significantly inhibited xenograft tumor growth in mice. In addition, the body weight of the experimental group mice receiving HF treatment was not significantly different from that of the control group mice. H&E staining also revealed no significant histopathological alterations in the heart, liver, lung, and kidney tissues of HF treated BALB/c-nu mice, indicating that HF did not induce significant systemic toxicity at the tested doses.

## Conclusion

Data from our *in vitro*, in silico, and *in vivo* experiments showed that HF exerts antitumor effects against ESCC by inhibiting tumor growth and promoting apoptosis through the PI3K/AKT/mTOR signaling pathway. HF may also possibly inhibit metastasis by regulating MMPs. Overall, these findings reveal the underlying mechanisms by which HF inhibits the growth of human ESCC and provide new evidence supporting therapeutic potential of HF in ESCC.

## Data Availability Statement

The datasets presented in this study can be found in online repositories. The names of the repository/repositories and accession number(s) can be found in the article/**Supplementary Material**.

## Ethics Statement

The animal study was reviewed and approved by the Institutional Animal Care and Use Committee of The Second Affiliated Hospital of Harbin Medical University (SYDW2021-046).

## Author Contributions

JG, SZ, and LZ contributed to the conception and design of this research. JG and SZ performed the experiments. JG and SZ analyzed the results. JG, SZ, JW, PZ, TL, and LZ wrote the paper. All authors contributed to the article and approved the submitted version.

## Conflict of Interest

The authors declare that the research was conducted in the absence of any commercial or financial relationships that could be construed as a potential conflict of interest.

## Publisher’s Note

All claims expressed in this article are solely those of the authors and do not necessarily represent those of their affiliated organizations, or those of the publisher, the editors and the reviewers. Any product that may be evaluated in this article, or claim that may be made by its manufacturer, is not guaranteed or endorsed by the publisher.
