# Corrigendum: Hinokiflavone Inhibits Growth of Esophageal Squamous Cancer By Inducing Apoptosis *via* Regulation of the PI3K/AKT/mTOR Signaling Pathway

**DOI:** 10.3389/fonc.2022.970020

**Published:** 2022-07-05

**Authors:** Jida Guo, Shengqiang Zhang, Jun Wang, Pengfei Zhang, Tong Lu, Linyou Zhang

**Affiliations:** Department of Thoracic Surgery, The Second Affiliated Hospital of Harbin Medical University, Harbin Medical University, Harbin, China

**Keywords:** esophageal cancer, hinokiflavone, apoptosis, KEGG analysis, molecular docking, PI3K/AKT/mTOR signal pathway

In the published article, there was an error in **Figures 6B**, **7G** as published. In **Figure 6B**, the image of Group 20 μM (cells treated with 20 μM of HF) of Transwell invasion assay was wrongly used in the original article during the figure assembly process. In **Figure 7G**, we recognized by ourselves that the H&E staining images of heart, liver, and spleen tissues of nude mice in Group 50 mg/kg were misused. The corrected **Figures 6**, **7** appear below.

**Figure 6:**
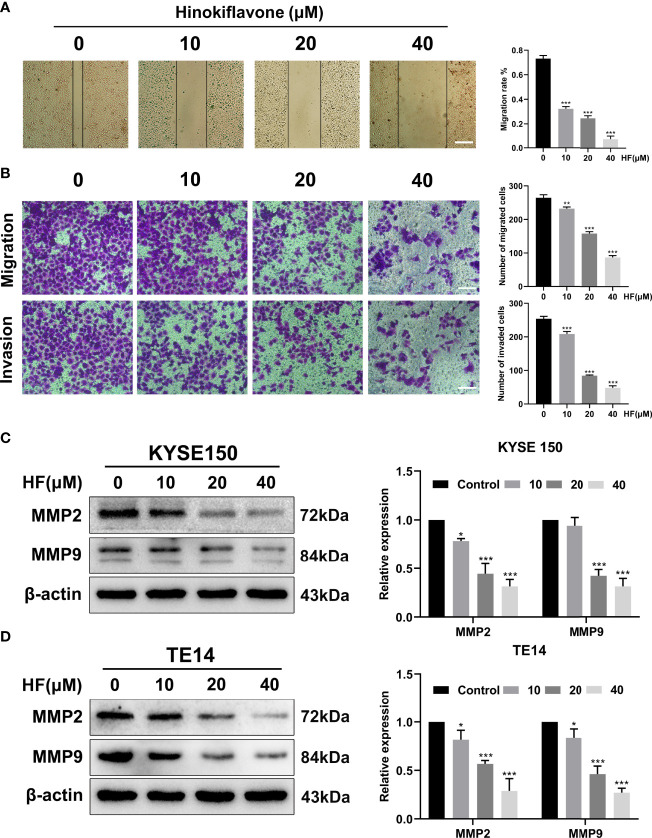
HF inhibits ESCC cell migration and invasion. **(A)** Cell migration was measured in a scratch-wound assay. KYSE150 cells were cultured until reaching approximately 80-90% cell density, the culture was scratched as described in Methods, and further cultured with various concentrations of HF (0, 10, 20, and 40 μM) for 24 h, after which the cells were fixed and photographed. Magnification, ×100; scale bars = 100 µm. The distance of cell migration from the same region was quantified using ImageJ. **(B)** KYSE150 cells were placed in the top chamber on the Transwell membrane with serum-free medium and the upper surface of the Transwell membrane was coated with/without Matrigel. Cells were treated using HF with a concentration gradient for 24 h, and then the cells were fixed and photographed using a microscope after crystal violet staining. Magnification, ×100; scale bars = 100 µm. Migrated and invaded cells were counted as described in the Methods. **(C)**, **(D)** KYSE150 cells and TE14 cells were treated with various concentrations of HF for 24 h, and then the cells were collected for protein extraction. The extracted proteins were later used for western blot analysis to determine the protein expression levels of MMP2 and MMP9. All the above experimental data are presented as the mean ± SD of three independent experiments. *P < 0.05; **P < 0.01; ***P < 0.001 compared to 0 μM vehicle group.

**Figure 7:**
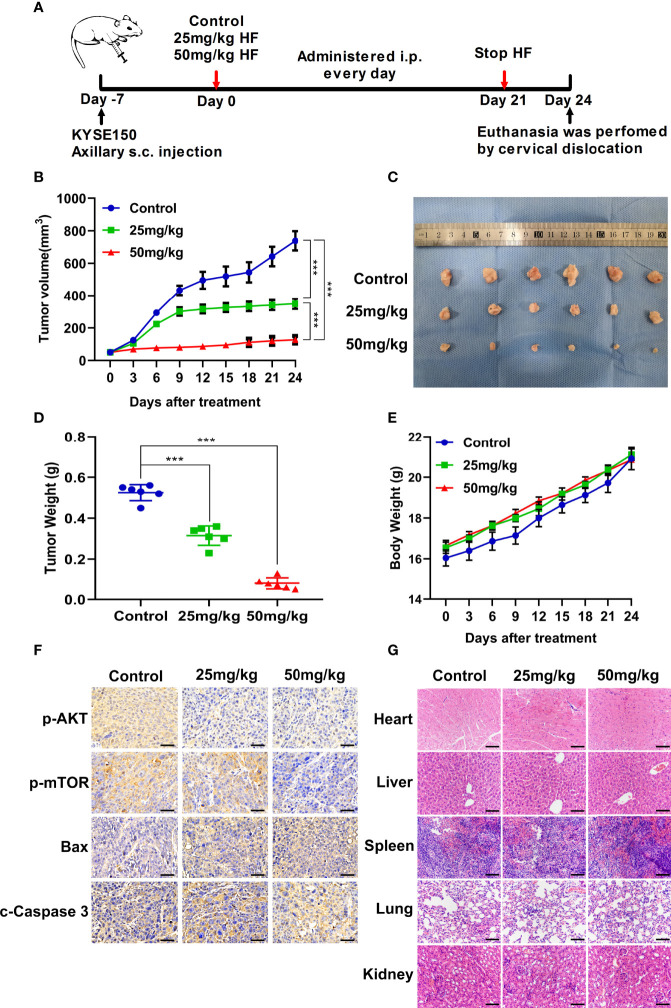
HF suppresses the growth of mouse ESCC xenograft tumors. **(A)** BALB/c-nu mice implanted with KYSE150 xenograft tumors were treated daily with an equal volume of saline (control group) or HF (25 or 50 mg/kg) by intraperitoneal injection for total 21 days. **(B)** The tumor volume was measured every 3 days, and the difference in tumor volume between HF treated and control mice is shown. ***P < 0.001. **(C)** After euthanasia of mice, subcutaneous xenografts were removed and photographed. **(D)** The removed xenograft tumors were weighed and graphed for statistical analysis. ***P < 0.001 vs the control group. **(E)** Body weights of tumor-bearing mice were measured every 3 days in the HF-treated and control groups. **(F)** Expression levels of p-AKT, p-mTOR, Bax, and cleaved-Caspase 3 were detected by immunohistochemistry in the xenograft tumors. Magnification, ×400; scale bars = 50 µm. **(G)** H&E staining of the heart, liver, spleen, lung, and kidney of experimental mice shows no pathological changes in the organ tissues of any group. Magnification, ×200; scale bars = 100 µm.

The authors apologize for these errors and state that this does not change the scientific conclusions of the article in any way. The original article has been updated.

## Publisher’s Note

All claims expressed in this article are solely those of the authors and do not necessarily represent those of their affiliated organizations, or those of the publisher, the editors and the reviewers. Any product that may be evaluated in this article, or claim that may be made by its manufacturer, is not guaranteed or endorsed by the publisher.

