# Supplementary material for: Hinokiflavone Inhibits Growth of Esophageal Squamous Cancer By Inducing Apoptosis via Regulation of the PI3K/AKT/mTOR Signaling Pathway
Source: Front Oncol. 2022 Feb 1;12:833719. doi: 10.3389/fonc.2022.833719 (PMC8844566; doi:10.3389/fonc.2022.833719)

Figure 1B

For KYSE150 cells

KYSE150 CCK-8 24h (OD value)

Blank wells	Control wells	HF 2.5μM	HF 5μM	HF 10μM	HF 20μM	HF 40μM	HF 80μM
0.202	0.872	0.87	0.796	0.743	0.621	0.52	0.485
0.181	0.837	0.828	0.779	0.771	0.647	0.531	0.512
0.19	1.077	0.985	0.975	0.89	0.819	0.567	0.553

KYSE150 CCK-8 24h (Cell viability)

Control groups	HF 2.5μM	HF 5μM	HF 10μM	HF 20μM	HF 40μM	HF 80μM
1	0.997	0.882	0.807	0.625	0.475	0.422
1	0.986	0.912	0.899	0.71	0.534	0.505
1	0.896	0.885	0.884	0.709	0.425	0.414

KYSE150 CCK-8 48h (OD value)

Blank wells	Control wells	HF 2.5μM	HF 5μM	HF 10μM	HF 20μM	HF 40μM	HF 80μM
0.22	0.77	0.756	0.658	0.592	0.568	0.445	0.384
0.189	0.819	0.791	0.756	0.69	0.632	0.475	0.471
0.281	0.874	0.841	0.731	0.76	0.619	0.49	0.46

KYSE150 CCK-8 48h (Cell viability)

Control groups	HF 2.5μM	HF 5μM	HF 10μM	HF 20μM	HF 40μM	HF 80μM
1	0.975	0.796	0.676	0.633	0.409	0.298
1	0.956	0.900	0.795	0.703	0.454	0.448
1	0.944	0.759	0.808	0.570	0.352	0.302

For TE14 cells

TE14 CCK-8 24h (OD value)

Blank wells	Control wells	HF 2.5μM	HF 5μM	HF 10μM	HF 20μM	HF 40μM	HF 80μM
0.127	0.825	0.756	0.745	0.672	0.66	0.572	0.463
0.103	0.829	0.802	0.771	0.643	0.629	0.541	0.406
0.119	0.807	0.798	0.759	0.65	0.592	0.528	0.45

TE14 CCK-8 24h (Cell viability)

Control groups	HF 2.5μM	HF 5μM	HF 10μM	HF 20μM	HF 40μM	HF 80μM
1	0.901	0.885	0.785	0.764	0.638	0.481
1	0.963	0.92	0.744	0.725	0.603	0.417
1	0.987	0.918	0.762	0.679	0.587	0.475

TE14 CCK-8 48h (OD value)

Blank wells	Control wells	HF 2.5μM	HF 5μM	HF 10μM	HF 20μM	HF 40μM	HF 80μM
0.195	1.07	0.939	0.817	0.71	0.629	0.578	0.388
0.177	0.908	0.775	0.811	0.706	0.655	0.557	0.458
0.207	1.061	0.959	0.913	0.7	0.685	0.599	0.387

TE14 CCK-8 48h (Cell viability)

Control groups	HF 2.5μM	HF 5μM	HF 10μM	HF 20μM	HF 40μM	HF 80μM
1	0.85	0.836	0.661	0.496	0.438	0.221
1	0.791	0.867	0.724	0.654	0.52	0.384
1	0.871	0.827	0.577	0.56	0.459	0.211

Figure 1C

For KYSE150 cells

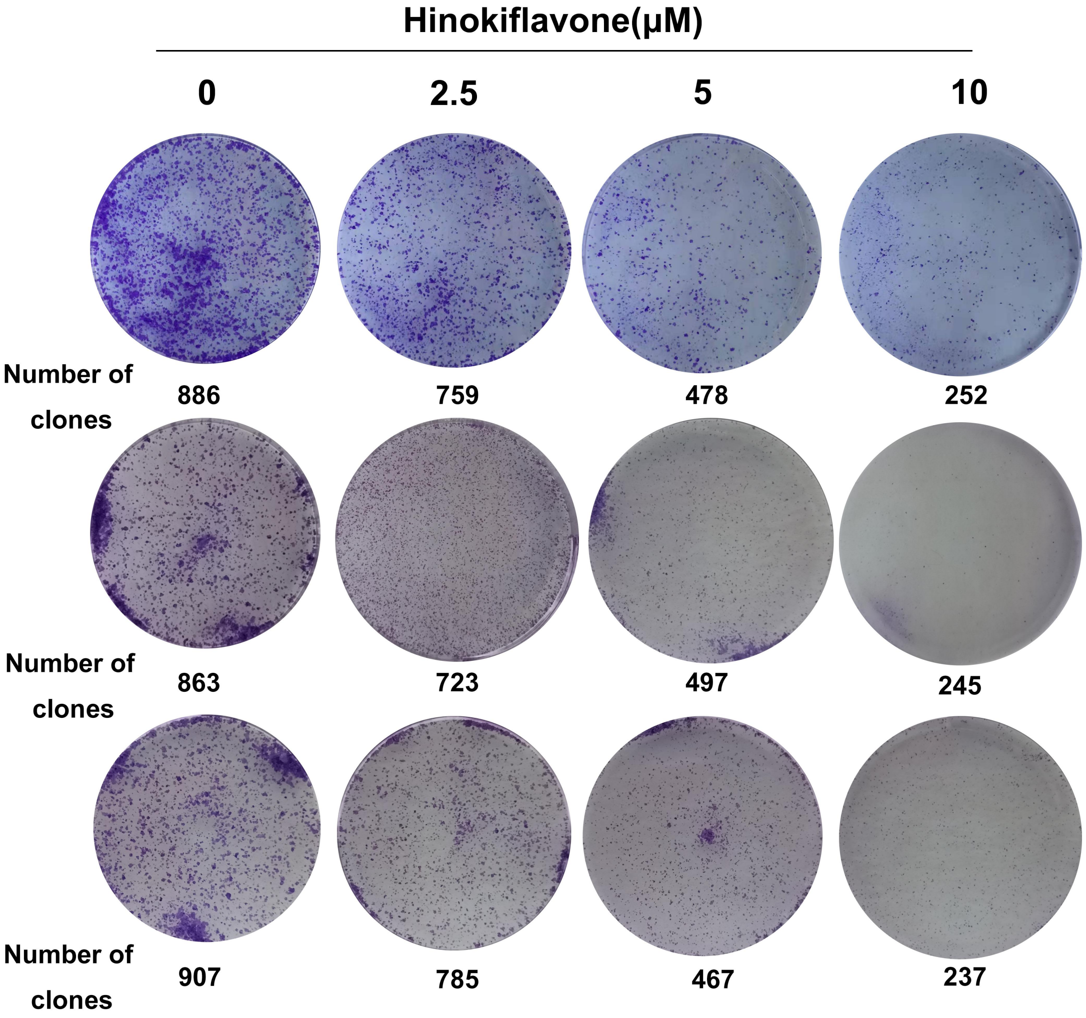

Colony forming number (%) for KYSE150			
HF 0μM	HF 2.5μM	HF 5μM	HF 10μM
1	83.8	57.6	28.4
1	86.5	51.5	26.1
1	85.7	54	28.4

For TE14 cells

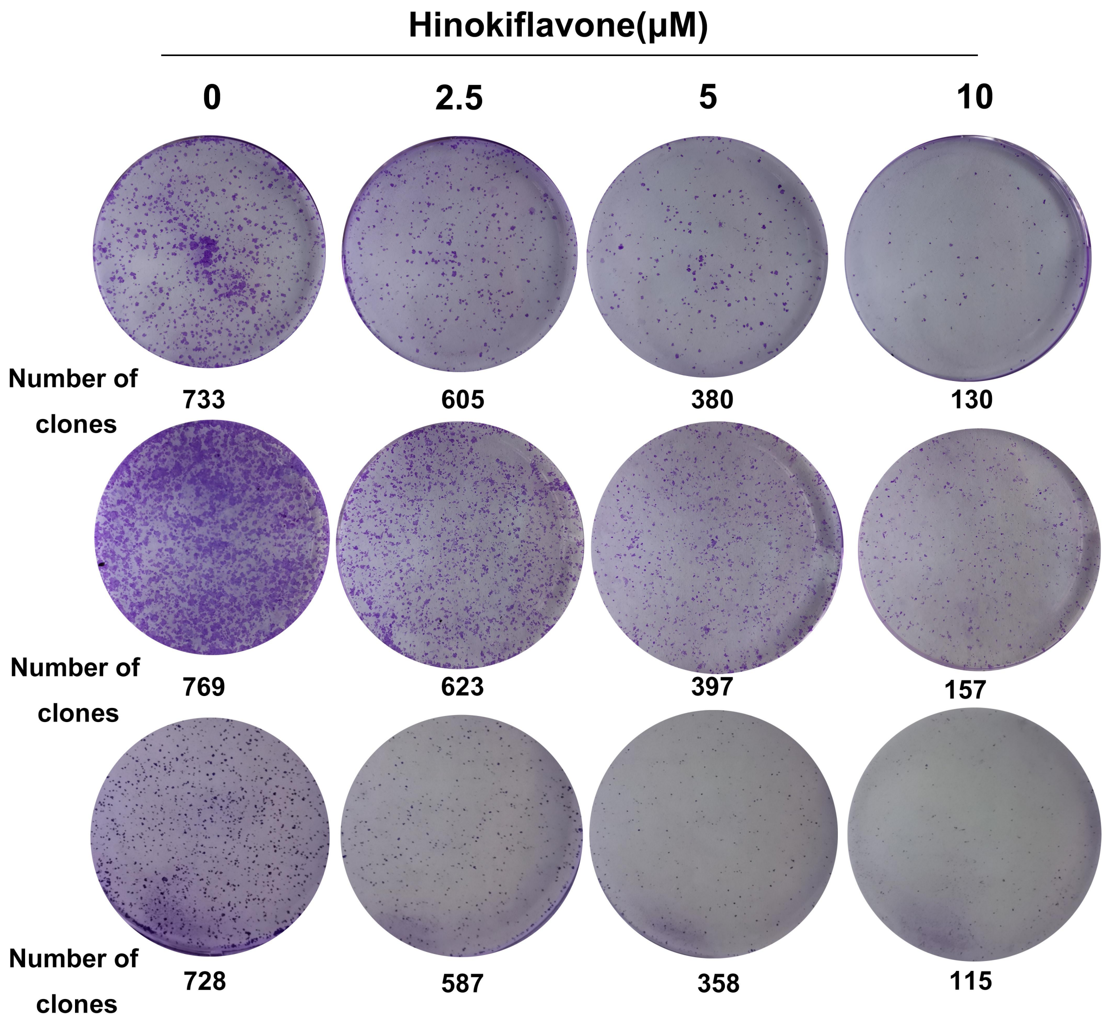

Colony forming number (%) for TE14			
HF 0μM	HF 2.5μM	HF 5μM	HF 10μM
1	81	51.6	20.4
1	80.6	49.2	15.8
1	82.5	51.8	17.7

Figure 1D

For KYSE150 cells

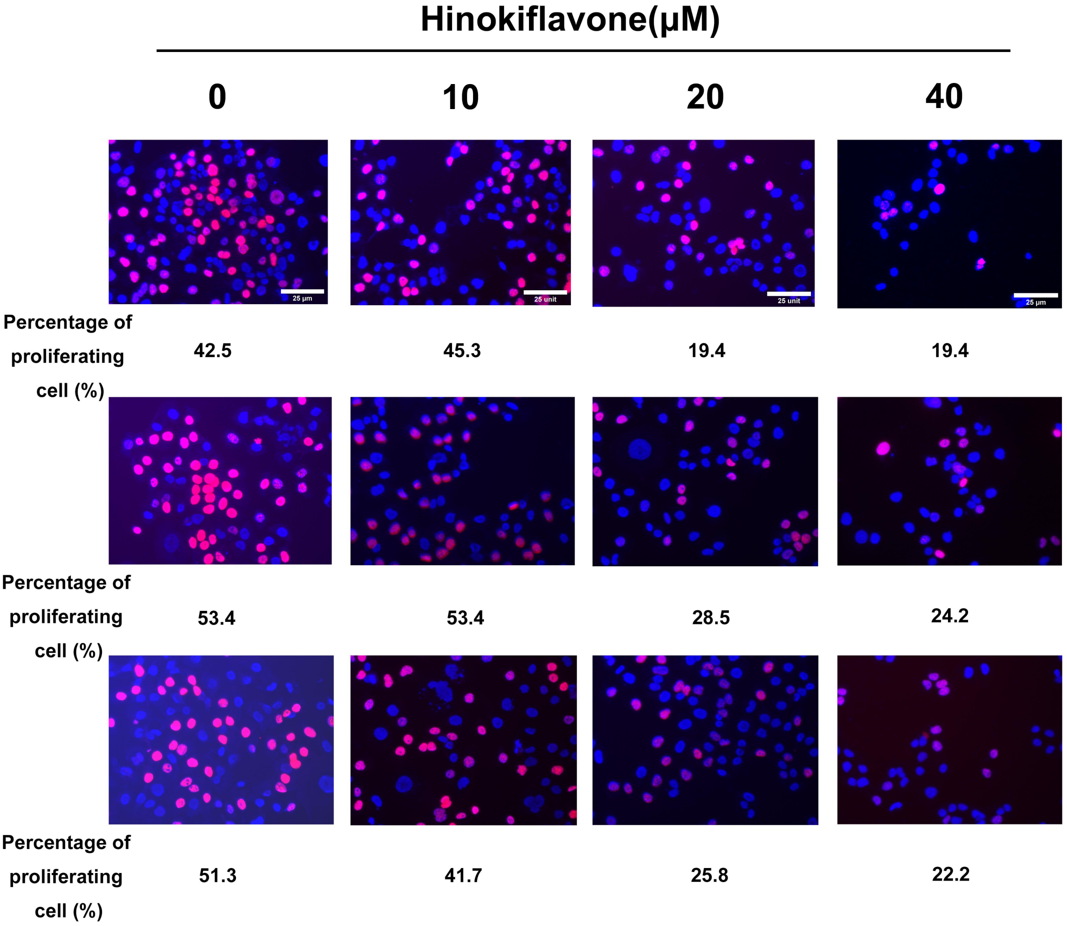

Percentage of proliferating cell (%)			
HF 0μM	HF 10μM	HF 20μM	HF 40μM
42.5	45.3	19.4	19.4
53.4	53.4	28.5	24.2
51.3	41.7	25.8	22.2

For TE14 cells

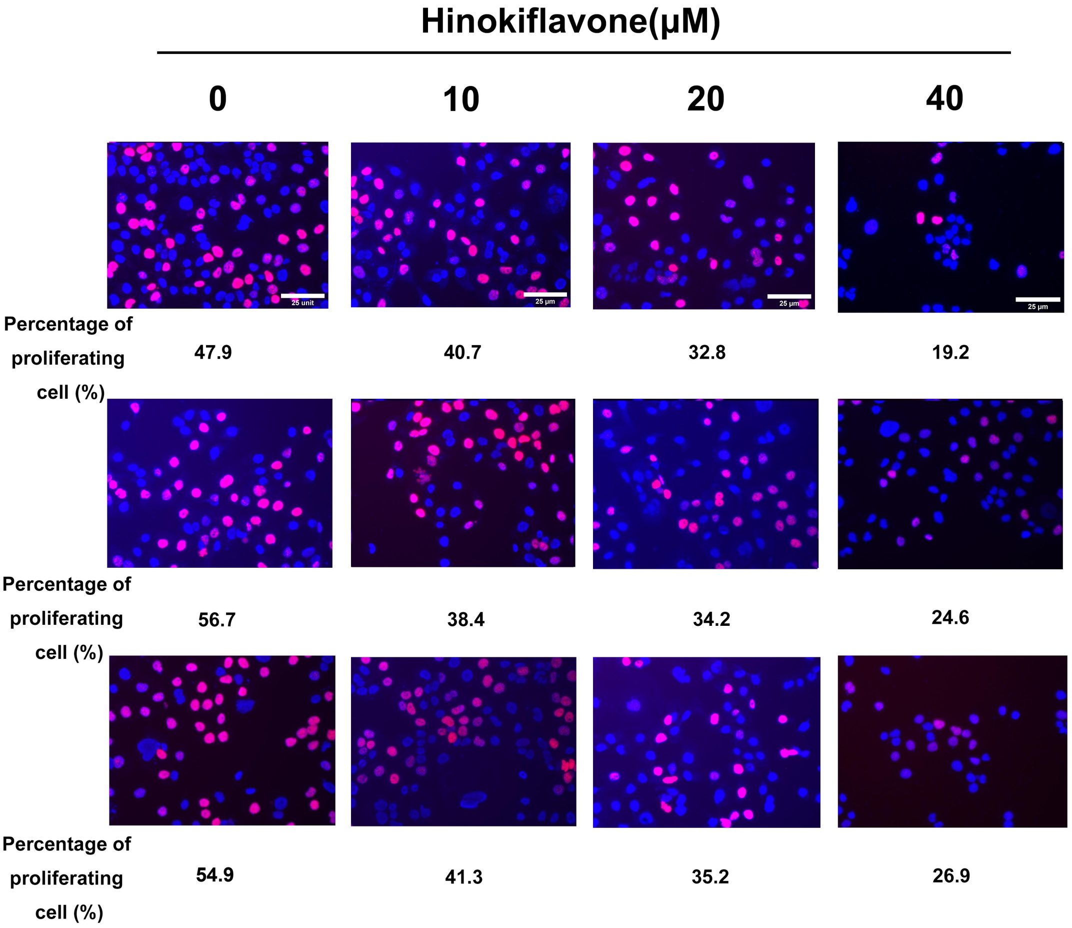

Percentage of proliferating cell (%)			
HF 0μM	HF 10μM	HF 20μM	HF 40μM
47.9	40.7	32.8	19.2
56.7	38.4	34.2	24.6
54.9	41.3	35.2	26.9

Figure 2A

For KYSE150 cells

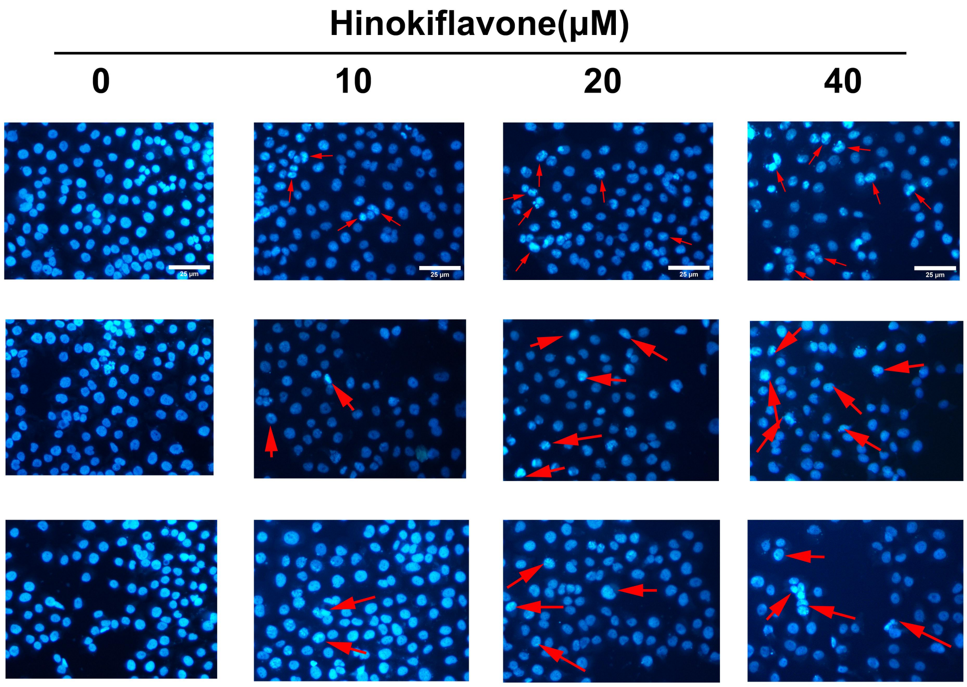

For TE14 cells

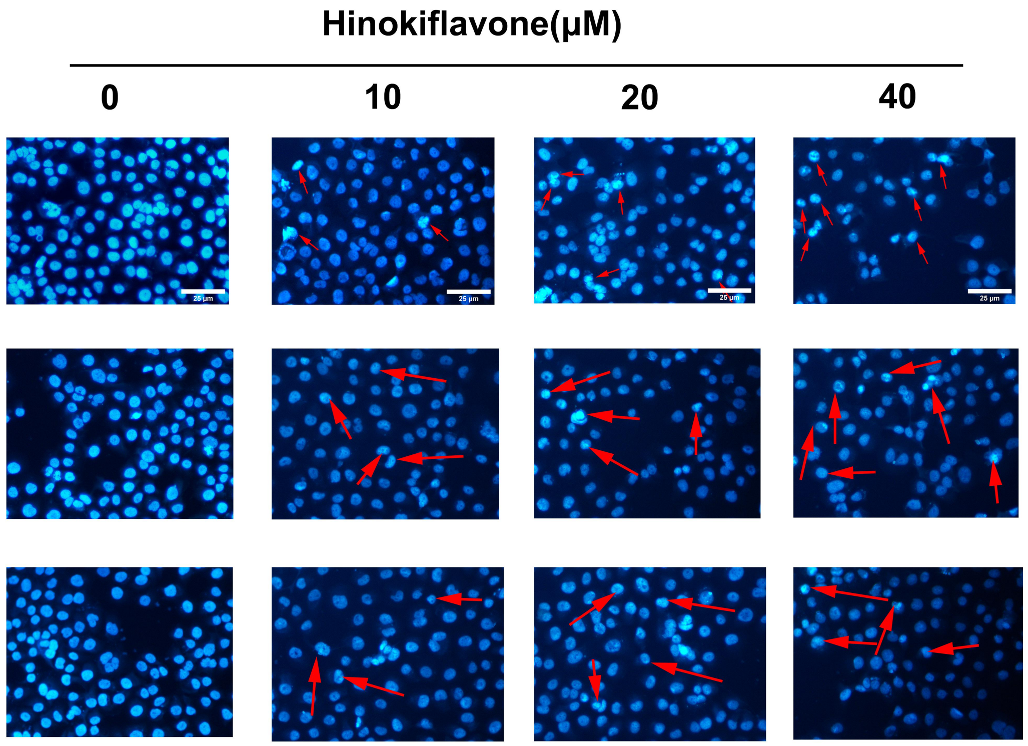

Figure 2B

For KYSE150 cells

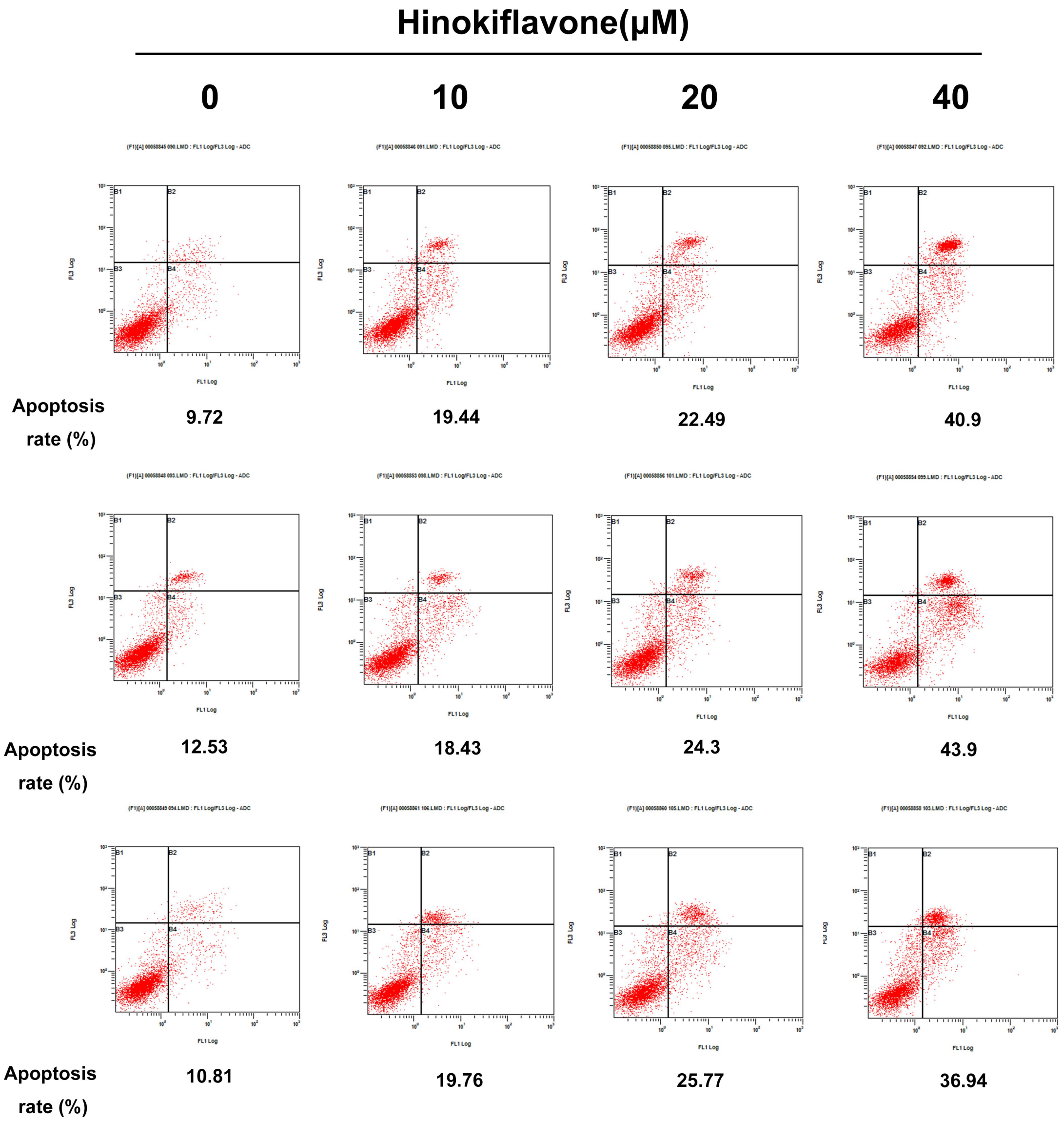

Apoptosis rate (%)			
HF 0μM	HF 10μM	HF 20μM	HF 40μM
9.72	19.44	22.49	40.9
12.53	18.43	24.3	43.9
10.81	19.76	25.77	36.94

For TE14 cells

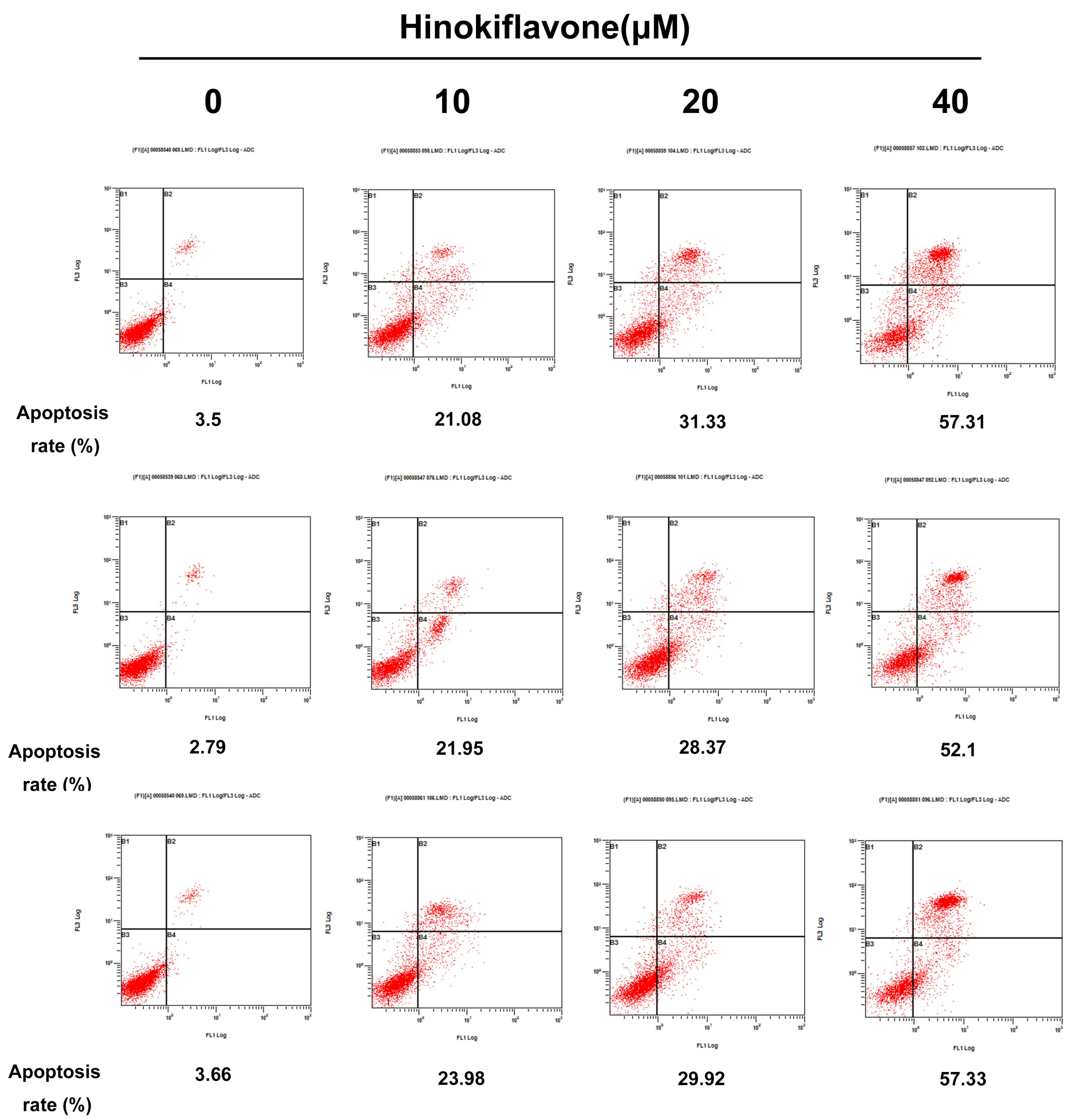

Apoptosis rate (%)			
HF 0μM	HF 10μM	HF 20μM	HF 40μM
3.5	21.08	31.33	57.31
2.79	21.95	28.37	52.1
3.66	23.98	29.92	57.33

Figure 2C

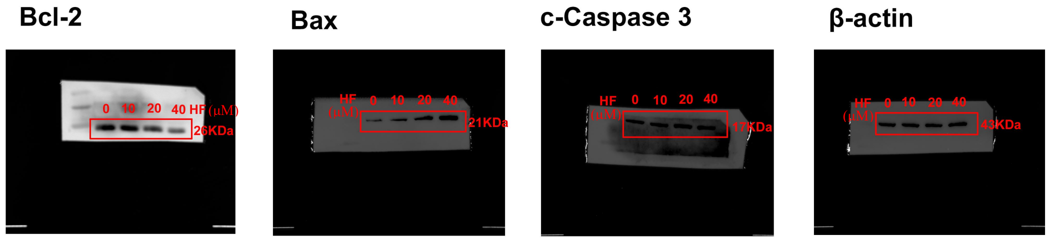

Protein quantification				
	Bcl-2	Bax	c-Caspase3	β-actin
HF 0μM	43790.894	12339.702	13150.004	32561.803
HF 10μM	39910.217	16293.459	15243.589	35938.238
HF 20μM	26127.409	25852.773	22315.48	35481.652
HF 40μM	14537.246	30849.48	32973.409	39547.359

Relative expression			
	Bcl-2	Bax	c-Caspase3
HF 0μM	1	1	1
HF 10μM	0.911381645	1.320409439	1.159207936
HF 20μM	0.596640228	2.095088925	1.696994161
HF 40μM	0.33196961	2.500018234	2.507482811

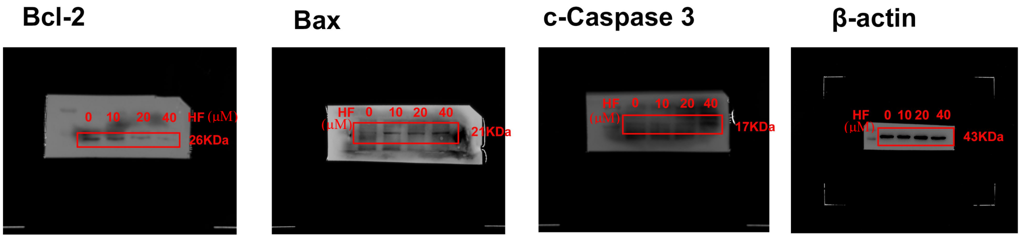

Protein quantification				
	Bcl-2	Bax	c-Caspase3	β-actin
HF 0μM	40686.324	10895.392	15146.681	32252.53
HF 10μM	30686.324	15521.874	19761.338	35202.874
HF 20μM	25318.924	20282.141	28581.501	35481.187
HF 40μM	13077.619	30282.141	38851.803	39547.359

Relative expression			
	Bcl-2	Bax	c-Caspase3
HF 0μM	1	1	1
HF 10μM	0.754217166	1.424627402	1.304664566
HF 20μM	0.622295688	1.861533848	1.886981115
HF 40μM	0.321425425	2.779353051	2.565037383

Bcl-2 Bax c-Caspase 3 β-actin

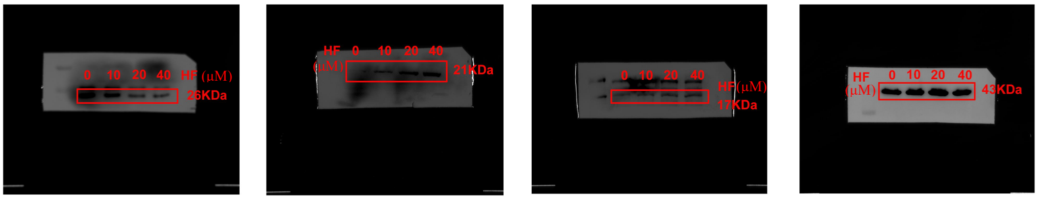

Protein quantification				
	Bcl-2	Bax	c-Caspase3	β-actin
HF 0μM	43719.459	13512.589	21041.995	32252.53
HF 10μM	28350.338	16029.782	25458.279	34730.681
HF 20μM	14640.439	22399.702	29301.773	35481.551
HF 40μM	10288.803	34987.874	49017.874	39434.581

Relative expression			
	Bcl-2	Bax	c-Caspase3
HF 0μM	1	1	1
HF 10μM	0.648460403	1.186285027	1.209879529
HF 20μM	0.334872373	1.657691357	1.39253778
HF 40μM	0.235336924	2.589279819	2.329525979

Figure 2D

Bcl-2 Bax c-Caspase 3 β-actin

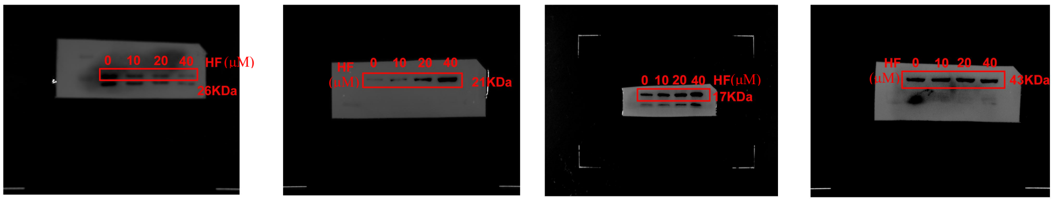

Protein quantification				
	Bcl-2	Bax	c-Caspase3	β-actin
HF 0μM	39172.823	13086.64	18322.066	37710.551
HF 10μM	21805.196	18224.288	27474.288	36180.744
HF 20μM	15146.317	22740.652	29551.459	39196.187
HF 40μM	5903.782	41920.581	46335.773	36620.066

Relative expression			
	Bcl-2	Bax	c-Caspase3
HF 0μM	1	1	1
HF 10μM	0.556640914	1.392587249	1.499519104
HF 20μM	0.386653701	1.737699822	1.612889016
HF 40μM	0.15071117	3.20331124	2.528960053

Bcl-2

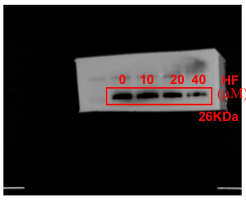

Bax

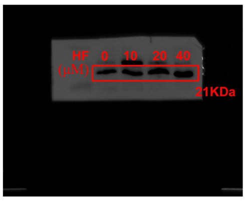

c-Caspase 3

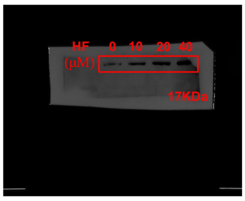

β-actin

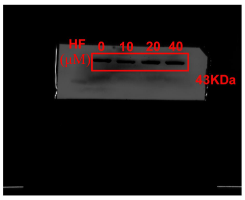

Protein quantification				
	Bcl-2	Bax	c-Caspase3	β-actin
HF 0μM	43097.459	14255.459	15442.69	39121.543
HF 10μM	31541.217	21813.459	18005.116	39683.756
HF 20μM	22322.53	28693.551	32439.995	38719.158
HF 40μM	15300.602	43744.53	38173.794	37203.38

Relative expression			
	Bcl-2	Bax	c-Caspase3
HF 0μM	1	1	1
HF 10μM	0.731857927	1.530182858	1.165931324
HF 20μM	0.517954666	2.012811443	2.100669961
HF 40μM	0.355023297	3.06861603	2.471965312

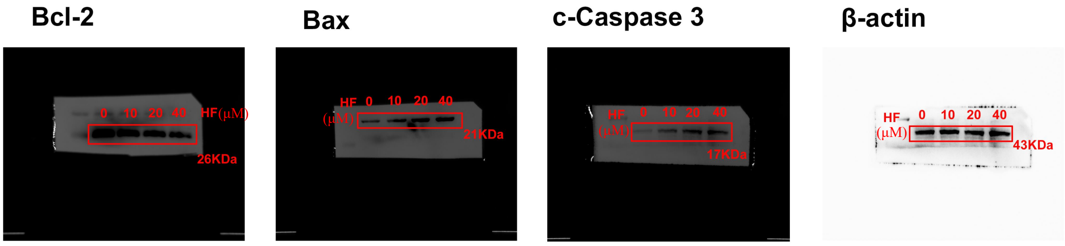

Protein quantification				
	Bcl-2	Bax	c-Caspase3	β-actin
HF 0μM	45484.48	8972.146	13028.894	36169.016
HF 10μM	38599.258	15884.945	13549.702	39065.12
HF 20μM	29345.116	20884.238	22227.693	38719.158
HF 40μM	17526.187	30264.187	37299.48	39209.673

Relative expression			
	Bcl-2	Bax	c-Caspase3
HF 0μM	1	1	1
HF 10μM	0.848624806	1.770473307	1.039973309
HF 20μM	0.64516767	2.327674784	1.706030688
HF 40μM	0.385322356	3.373126897	2.862827804

Figure 5A-C

For KYSE150 cells

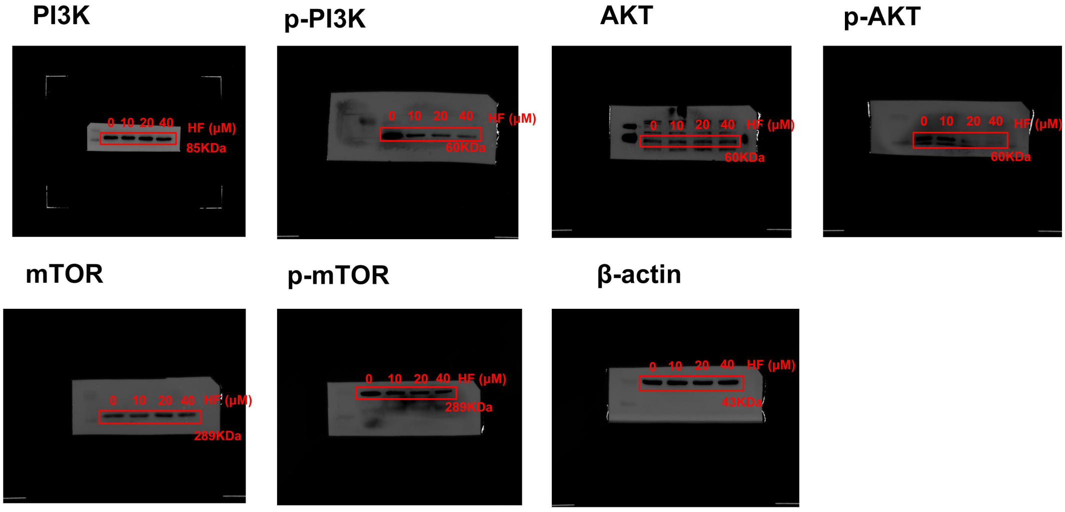

Protein quantification							
	PI3K	p-PI3K	AKT	p-AKT	mTOR	p-MTOR	β-actin
HF 0μM	39807.43	38373.602	36822.309	39493.409	40082.622	37804.48	36370.56
HF 10μM	41370.309	29781.489	35332.087	37564.652	38137.723	35149.673	37379.338
HF 20μM	39536.43	15703.347	35932.38	19856.338	37724.773	17305.459	37073.731
HF 40μM	41611.945	14190.619	36300.48	14494.912	39455.066	15122.388	36771.853

Relative expression						
	PI3K	p-PI3K	AKT	p-AKT	mTOR	p-MTOR
HF 0μM	1	1	1	1	1	1
HF 10μM	1.039260987	0.776093133	0.959529371	0.95116256	0.95147775	0.929775334
HF 20μM	0.993192226	0.409222647	0.975831798	0.502775995	0.94117528	0.722281037
HF 40μM	1.04533111	0.369801589	0.985828455	0.367021039	0.984343439	0.457762122

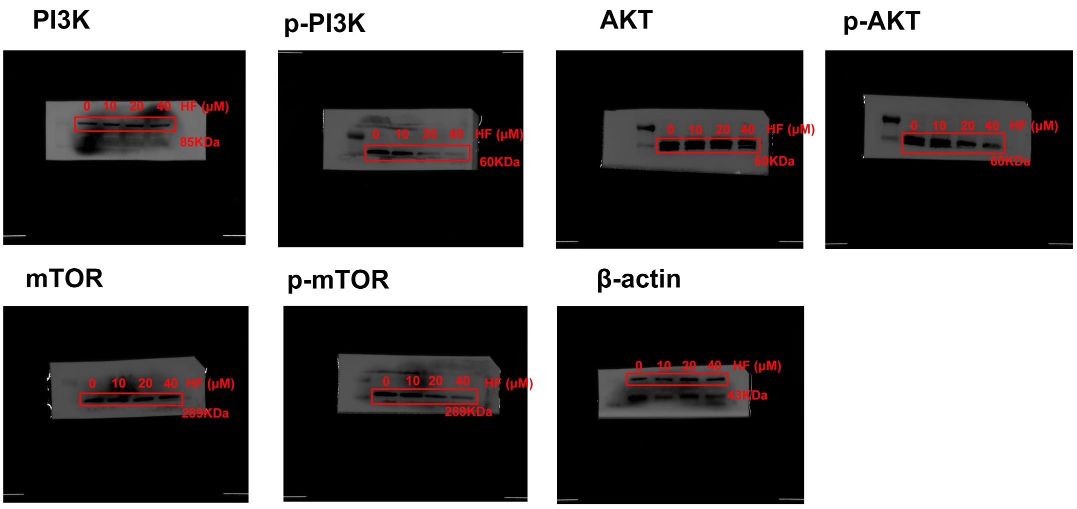

Protein quantification							
	PI3K	p-PI3K	AKT	p-AKT	mTOR	p-MTOR	β-actin
HF 0μM	32926.551	37903.924	38075.409	36291.066	28824.258	44807.238	35892.823
HF 10μM	33030.016	34317.794	38264.258	34794.016	28231.258	41852.551	36794.258
HF 20μM	33802.187	20400.995	36221.258	22493.681	27917.844	21556.782	36440.53
HF 40μM	34389.823	20105.581	37074.56	13806.317	27300.409	17187.991	35899.874

Relative expression						
	PI3K	p-PI3K	AKT	p-AKT	mTOR	p-MTOR
HF 0μM	1	1	1	1	1	1
HF 10μM	1.003142297	0.905388951	1.004959868	0.958748801	0.979427051	0.934057819
HF 20μM	1.026593614	0.538229103	0.951303189	0.619813179	0.968553779	0.481100442
HF 40μM	1.044440488	0.530435345	0.973714032	0.380432942	0.947133106	0.383598538

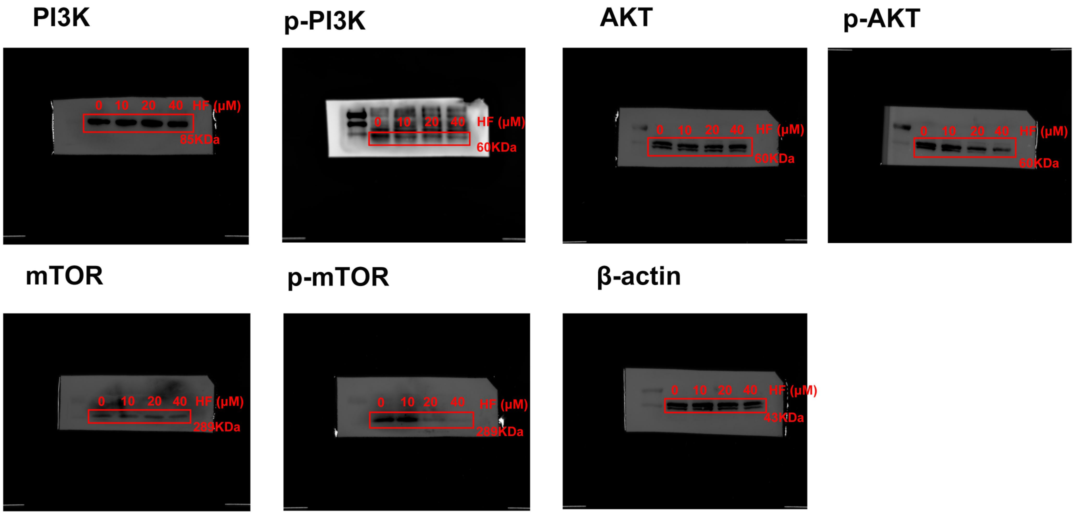

Protein quantification							
	PI3K	p-PI3K	AKT	p-AKT	mTOR	p-MTOR	β-actin
HF 0μM	42807.42	41938.945	37660.924	37216.095	37806.773	47885.874	37165.551
HF 10μM	41376.317	32337.894	38795.974	36619.894	37106.894	42358.631	37046.693
HF 20μM	42537.45	15831.388	39510.56	18624.045	36557.51	26398.874	36104.673
HF 40μM	41615.946	15204.912	39070.903	7289.711	38614.995	10820.489	36312.066

Relative expression						
	PI3K	p-PI3K	AKT	p-AKT	mTOR	p-MTOR
HF 0μM	1	1	1	1	1	1
HF 10μM	0.96656881	0.771070755	1.030138666	0.983980023	0.981488	0.884574666
HF 20μM	0.993693383	0.377486558	1.049112868	0.500429854	0.966956635	0.551287296
HF 40μM	0.972166648	0.362548748	1.037438779	0.195875225	1.021377704	0.225964112

For TE14 cells

PI3K

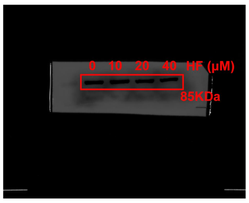

p-PI3K

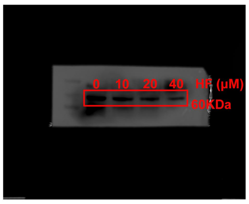

AKT

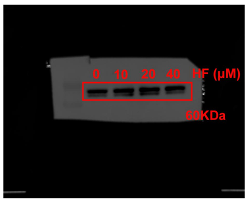

p-AKT

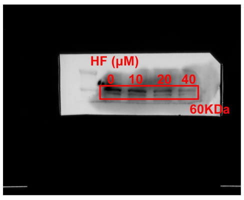

mTOR

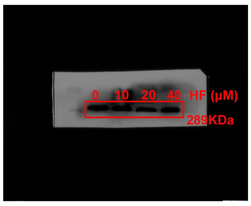

p-mTOR

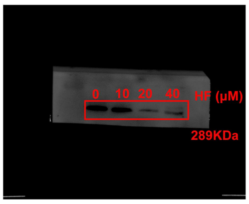

β-actin

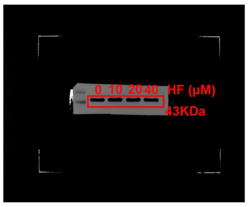

Protein quantification							
	PI3K	p-PI3K	AKT	p-AKT	mTOR	p-MTOR	β-actin
HF 0μM	32583.693	37766.602	30489.045	40924.459	33880.338	41456.48	38483.288
HF 10μM	31486.108	26513.723	29403.238	39169.924	34292.635	35316.543	38921.137
HF 20μM	30359.522	24687.53	32603.116	29850.602	33701.472	18805.957	38006.723
HF 40μM	28610.258	14023.095	29364.359	11259.782	34457.066	10410.409	39244.35

Relative expression						
	PI3K	p-PI3K	AKT	p-AKT	mTOR	p-MTOR
HF 0μM	1	1	1	1	1	1
HF 10μM	0.966314899	0.702041529	0.964386979	0.957127472	1.012169212	0.851894396
HF 20μM	0.931739751	0.653686821	1.069338708	0.72940737	0.994720655	0.453631302
HF 40μM	0.878054492	0.371309418	0.9631118	0.275135757	1.017022498	0.251116569

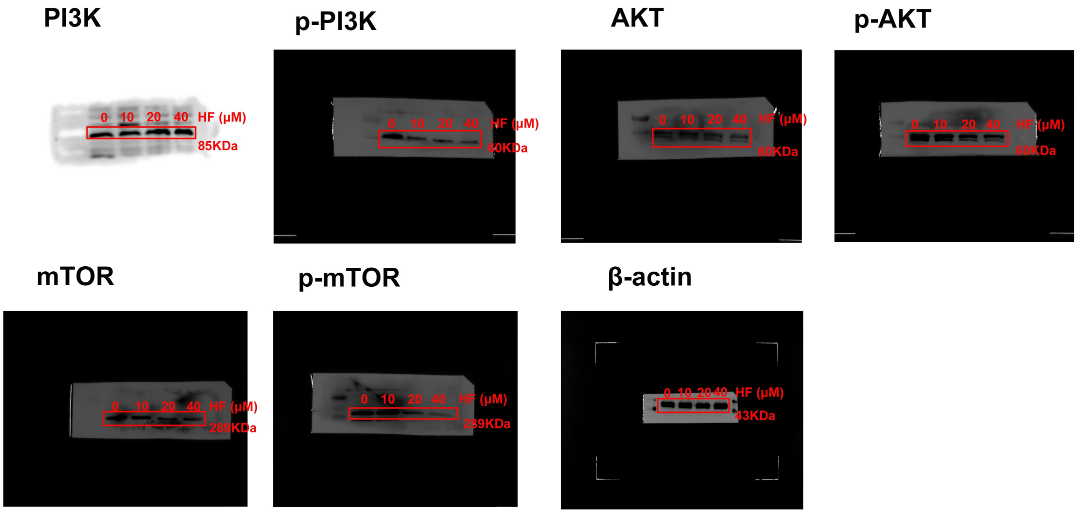

Protein quantification							
	PI3K	p-PI3K	AKT	p-AKT	mTOR	p-MTOR	β-actin
HF 0μM	38151.238	35366.137	32611.095	49970.066	36865.48	45043.229	37617.773
HF 10μM	39713.2	29681.844	33092.409	41588.652	36522.137	43555.179	36948.522
HF 20μM	39064.999	28355.723	34618.886	28342.844	35550.38	14283.53	36916.501
HF 40μM	38928.702	19829.551	33812.238	19736.175	36551.48	3196.167	37514.3

Relative expression						
	PI3K	p-PI3K	AKT	p-AKT	mTOR	p-MTOR
HF 0μM	1	1	1	1	1	1
HF 10μM	1.040941319	0.839273003	1.01475921	0.832271304	0.990686599	0.966963958
HF 20μM	1.023951018	0.801776089	1.061567727	0.567196449	0.964327062	0.317107151
HF 40μM	1.020378474	0.560693157	1.036832342	0.394959955	0.9643569	0.070957768

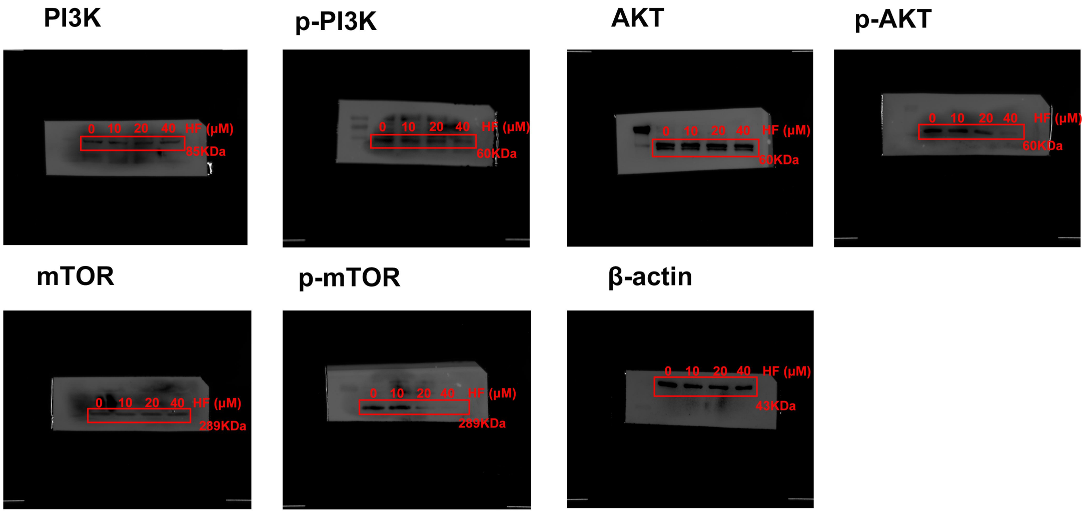

Protein quantification							
	PI3K	p-PI3K	AKT	p-AKT	mTOR	p-MTOR	β-actin
HF 0μM	34650.066	36042.016	43923.803	39382.338	30397.087	39877.401	37342.066
HF 10μM	35088.735	26133.116	41707.409	32900.045	31031.359	38989.442	36592.522
HF 20μM	35052.049	22990.459	43615.187	22405.924	32426.368	18031.522	36690.501
HF 40μM	35626.874	19923.045	43885.51	19442.61	33371.279	7012.974	37325.836

Relative expression						
	PI3K	p-PI3K	AKT	p-AKT	mTOR	p-MTOR
HF 0μM	1	1	1	1	1	1
HF 10μM	1.012659976	0.725073647	0.949540025	0.835401011	1.02086621	0.977732777
HF 20μM	1.011601219	0.360425427	0.992973832	0.568933312	1.066759061	0.452173952
HF 40μM	1.028190653	0.552772769	0.999128195	0.493688567	1.097844639	0.175863367

Figure 5D-E

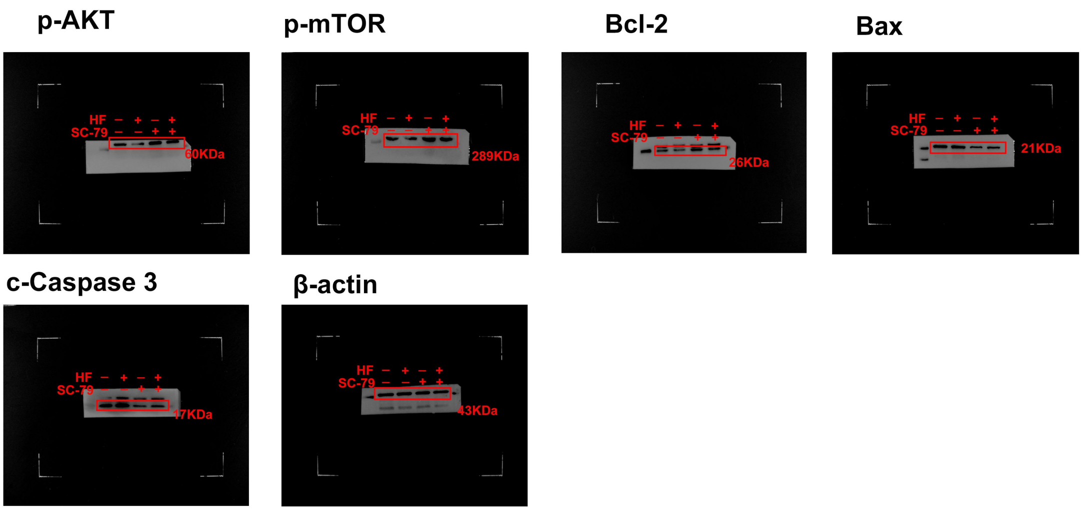

Protein quantification						
	p-AKT	p-MTOR	Bcl-2	Bax	c-Caspase3	β-actin
Control	29931.995	24647.752	33346.066	39803.066	32972.388	39566.48
HF	18402.238	12750.974	18593.924	40627.48	42261.208	39297.137
SC-79	34823.823	34683.844	39586.016	21966.631	9911.146	38181.723
HF+SC-79	25051.995	28247.116	30825.167	24990.773	21724.773	38184.966

Relative expression					
	p-AKT	p-MTOR	Bcl-2	Bax	c-Caspase3
Control	1	1	1	1	1
HF	0.614801586	0.517328071	0.557604726	1.020712324	1.281715113
SC-79	1.163431405	1.407180825	1.187127021	0.551882888	0.300589269
HF+SC-79	0.836963757	1.146032141	0.924401907	0.627860502	0.658877756

p-AKT

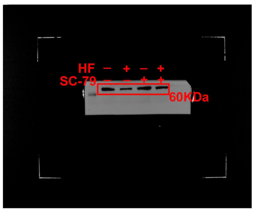

p-mTOR

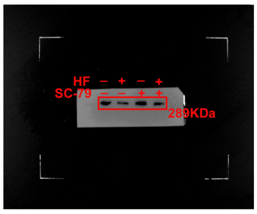

Bcl-2

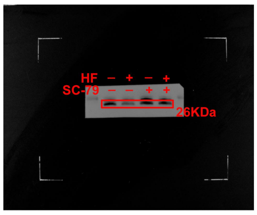

Bax

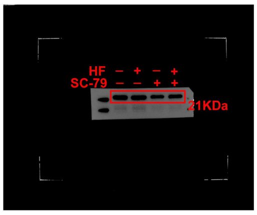

c-Caspase 3

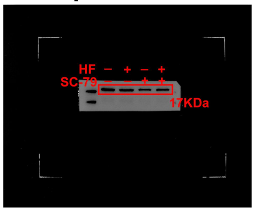

β-actin

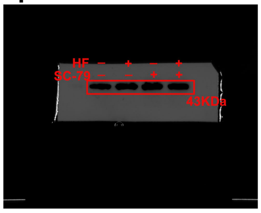

Protein quantification						
	p-AKT	p-MTOR	Bcl-2	Bax	c-Caspase3	β-actin
Control	35005.288	30867.581	28168.803	38302.066	31181.238	39226.602
HF	27941.045	18929.338	20613.53	42057.43	42714.238	38953.137
SC-79	41885.723	45441.409	35526.773	24869.874	19458.581	39764.43
HF+SC-79	29739.48	35273.137	26497.409	27922.894	22615.43	38642.572

Relative expression					
	p-AKT	p-MTOR	Bcl-2	Bax	c-Caspase3
Control	1	1	1	1	1
HF	0.798194976	0.613243325	0.731785799	1.098045991	1.369869856
SC-79	1.196554161	1.472140269	1.261209892	0.649308943	0.624047737
HF+SC-79	0.849571071	1.142724368	0.940665068	0.72901796	0.725289676

p-AKT

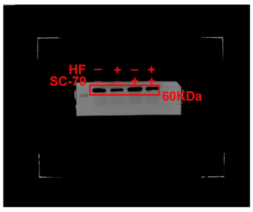

p-mTOR

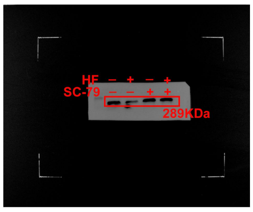

Bcl-2

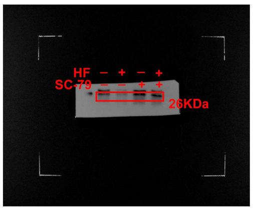

Bax

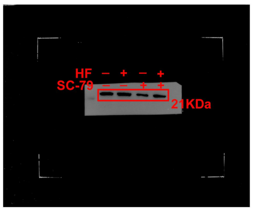

c-Caspase 3

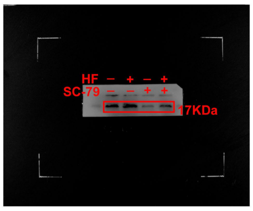

β-actin

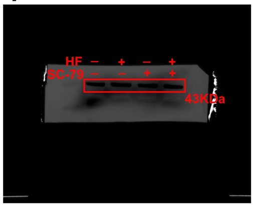

Protein quantification						
	p-AKT	p-MTOR	Bcl-2	Bax	c-Caspase3	β-actin
Control	25111.045	21364.53	23241.752	38894.652	38992.894	38844.551
HF	16762.702	17002.702	10969.388	42019.551	47809.894	38448.329
SC-79	32752.288	30925.137	32350.673	27107.995	20504.581	39376.966
HF+SC-79	28152.874	30370.581	26206.045	28809.48	24184.652	39420.501

Relative expression					
	p-AKT	p-MTOR	Bcl-2	Bax	c-Caspase3
Control	1	1	1	1	1
HF	0.667542988	0.795837868	0.471969067	1.080342639	1.226118123
SC-79	1.304298089	1.447499056	1.391920583	0.696959443	0.525854301
HF+SC-79	1.121135102	1.421542201	1.127541719	0.740705432	0.620232292

Figure 5F

KYSE150 CCK-8 24h (OD value)

Blank wells	Control	HF	SC-79	HF+SC-79
0.219	0.66	0.48	0.658	0.512
0.209	0.642	0.459	0.648	0.577
0.204	0.639	0.459	0.635	0.536
0.209	0.65	0.448	0.635	0.501

KYSE150 CCK-8 24h (Cell viability)			
Control	HF	SC-79	HF+SC-79
1	0.82	0.998	0.852
1	0.817	1.006	0.935
1	0.82	0.996	0.897

Figure 6A

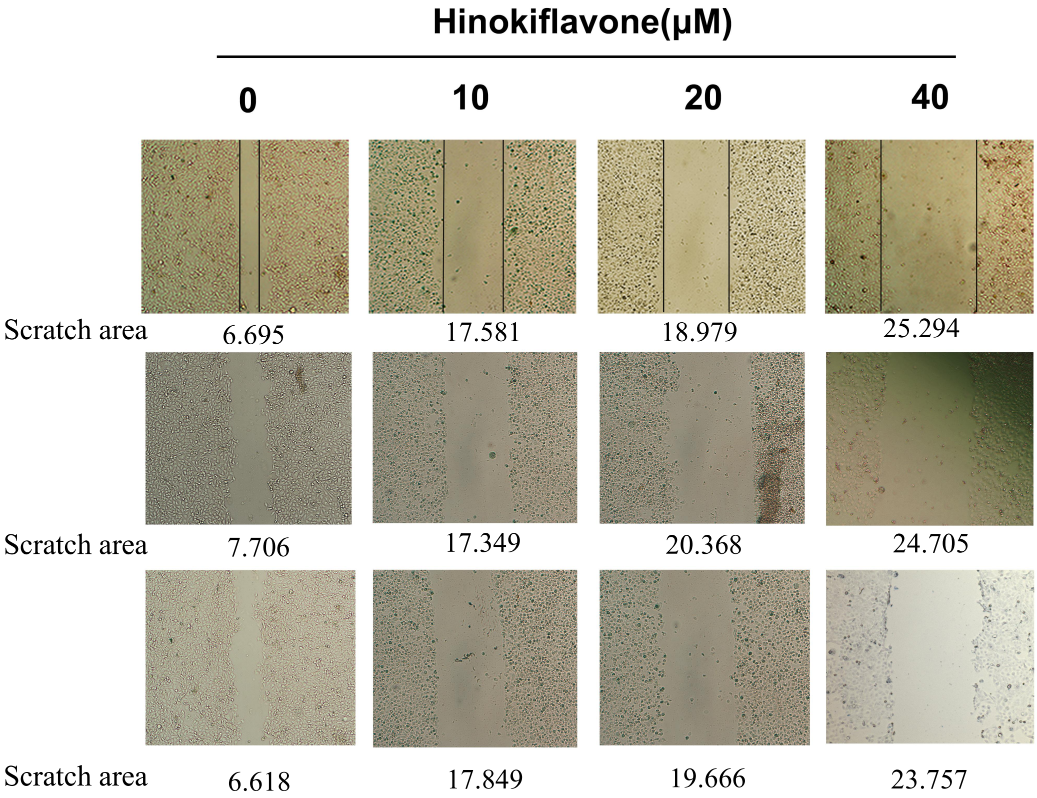

Scratch area

HF 0μM	HF 10μM	HF 20μM	HF 40μM	Control
6.695	17.581	18.979	25.294	25.901
7.706	17.349	20.368	24.705	26.367
6.618	17.849	19.666	23.757	25.74

Migration rate %

HF 0μM	HF 10μM	HF 20μM	HF 40μM
0.70248253	0.321223119	0.267248369	0.046175823
0.749004437	0.342018432	0.227519248	0.098987371
0.743783994	0.306565657	0.235975136	0.073427725

Figure 6B

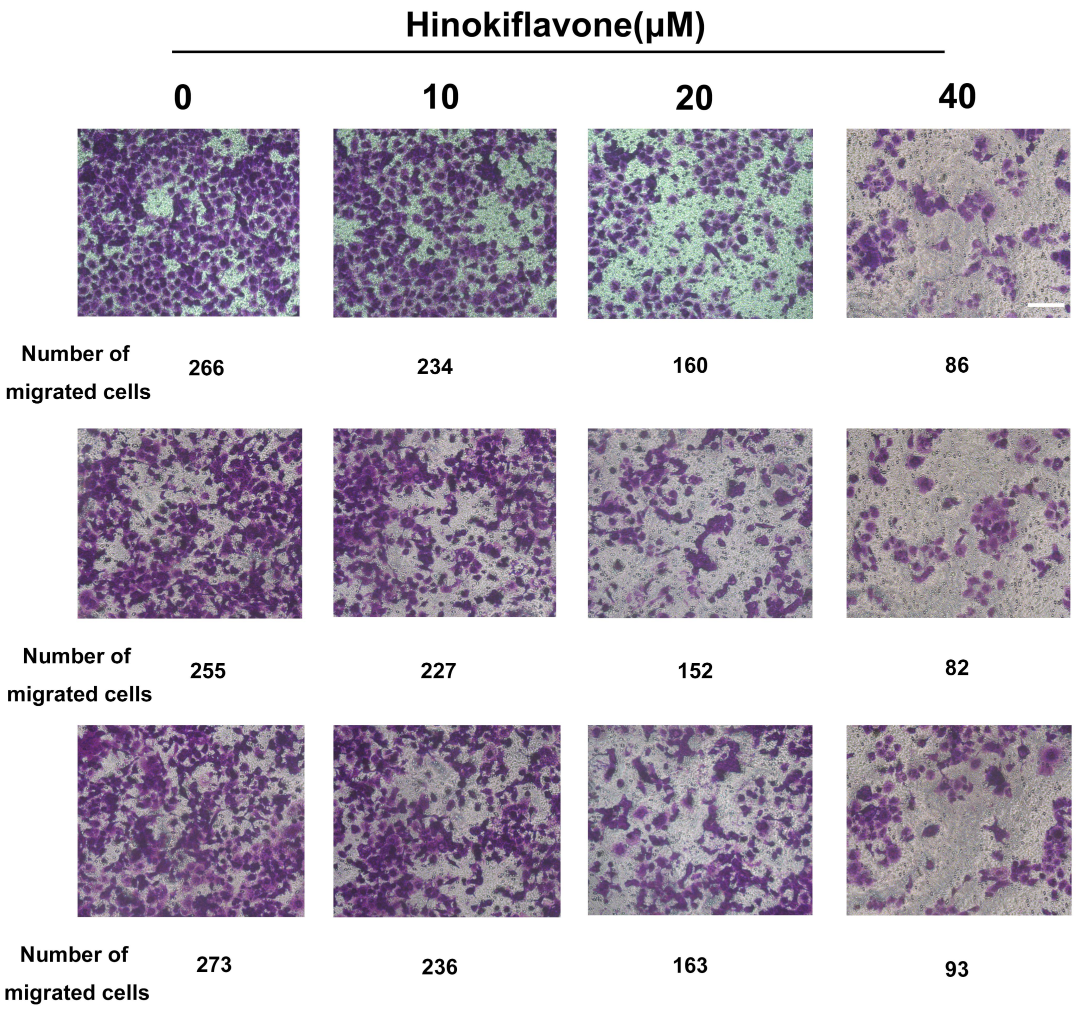

Number of migrated cells

HF 0μM	HF 10μM	HF 20μM	HF 40μM
266	234	160	86
255	227	152	82
273	236	163	93

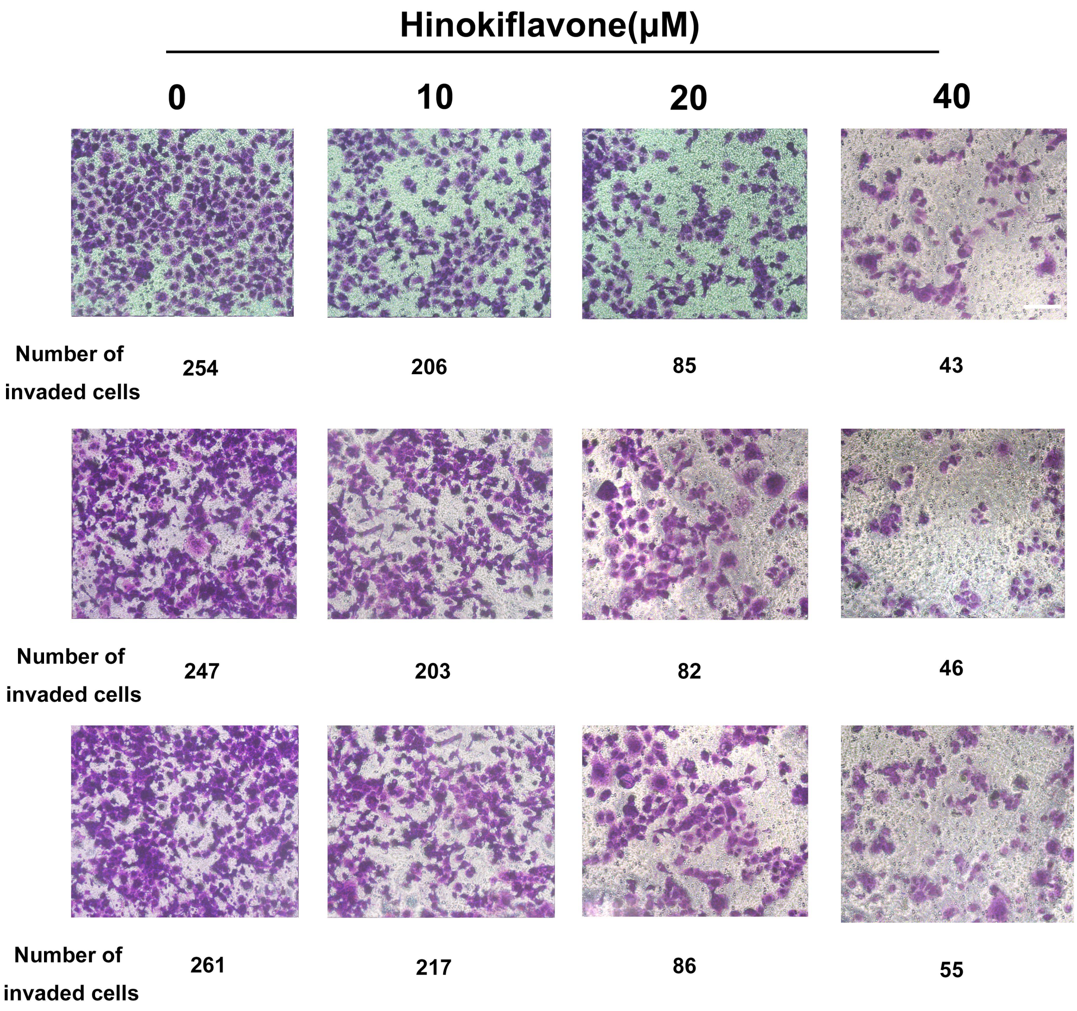

Number of invaded cells

HF 0μM	HF 10μM	HF 20μM	HF 40μM
254	206	85	43
247	203	82	46
261	217	86	55

Figure 6C

MMP2

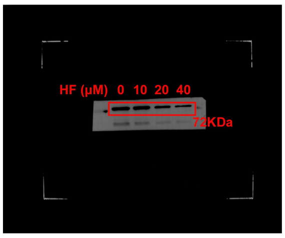

MMP9

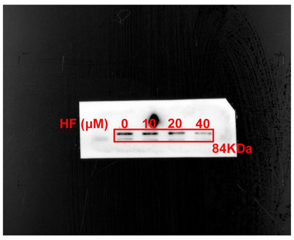

β-actin

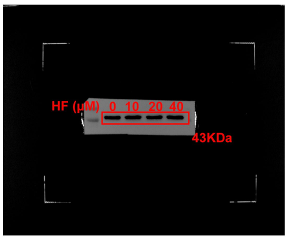

Protein quantification			
	MMP2	MMP9	β-actin
HF 0μM	41104.409	39366.045	38644.095
HF 10μM	32916.409	38893.43	39303.409
HF 20μM	22340.066	16908.66	37284.116
HF 40μM	15793.43	9754.368	39191.844

Relative expression		
	MMP2	MMP9
HF 0μM	1	1
HF 10μM	0.800799958	0.987994349
HF 20μM	0.543495614	0.429523972
HF 40μM	0.384227152	0.247786335

MMP2

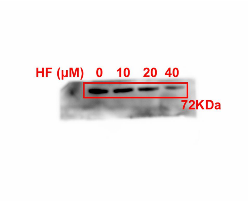

MMP9

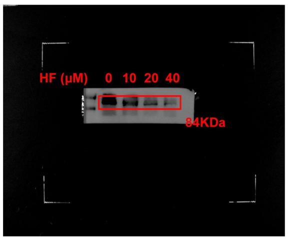

β-actin

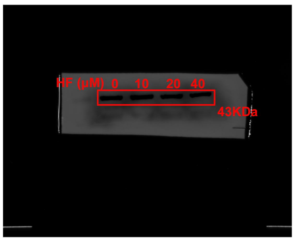

Protein quantification			
	MMP2	MMP9	β-actin
HF 0μM	40993.693	32447.602	37085.459
HF 10μM	32736.614	27362.602	38996.874
HF 20μM	18760.238	11563.51	36907.187
HF 40μM	12999.631	9268.56	38960.016

Relative expression		
	MMP2	MMP9
HF 0μM	1	1
HF 10μM	0.798576844	0.8432858
HF 20μM	0.457637178	0.356374872
HF 40μM	0.317112952	0.285646995

MMP2

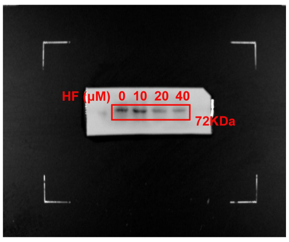

MMP9

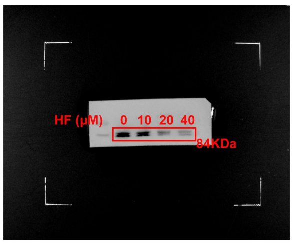

β-actin

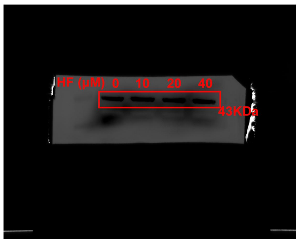

Protein quantification			
	MMP2	MMP9	β-actin
HF 0μM	39092.966	31079.765	37733.359
HF 10μM	29467.258	30853.158	38478.723
HF 20μM	12911.602	15069.48	36329.329
HF 40μM	9422.238	12676.066	38690.966

Relative expression		
	MMP2	MMP9
HF 0μM	1	1
HF 10μM	0.753773914	0.992708857
HF 20μM	0.330279417	0.48486467
HF 40μM	0.241021313	0.407855915

Figure 6D

MMP2

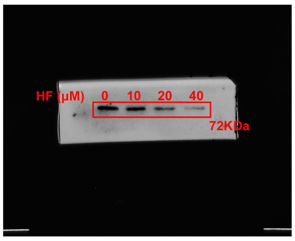

MMP9

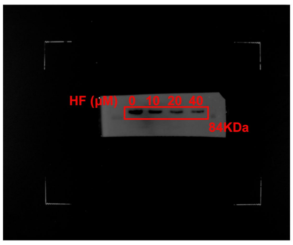

β-actin

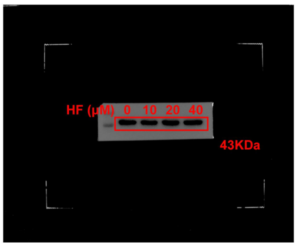

Protein quantification			
	MMP2	MMP9	β-actin
HF 0μM	39118.116	33662.844	37615.066
HF 10μM	27709.945	28122.087	38581.48
HF 20μM	23057.995	17960.673	39717.238
HF 40μM	7697.773	9884.752	40669.43

Relative expression		
	MMP2	MMP9
HF 0μM	1	1
HF 10μM	0. 708366042	0. 83540437
HF 20μM	0. 5894454438	0. 533545918
HF 40μM	0. 196782815	0. 293639836

MMP2

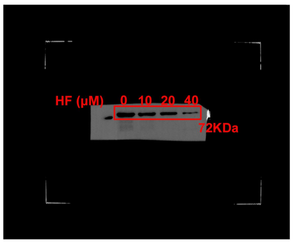

MMP9

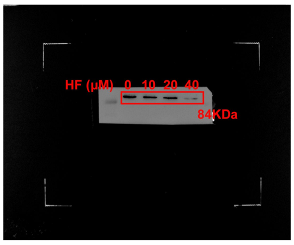

β-actin

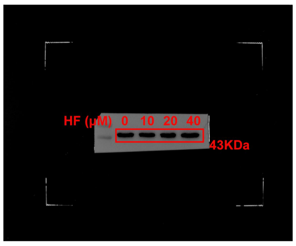

Protein quantification			
	MMP2	MMP9	β-actin
HF 0μM	30416.238	43849.137	38812.016
HF 10μM	25710.137	32704.217	35179.016
HF 20μM	16039.61	16343.782	39280.894
HF 40μM	13162.51	13296.51	40579.066

Relative expression		
	MMP2	MMP9
HF 0μM	1	1
HF 10μM	0.845276691	0.745834907
HF 20μM	0.527337076	0.372727564
HF 40μM	0.432746154	0.303233106

MMP2

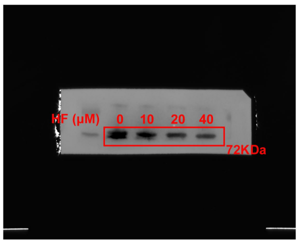

MMP9

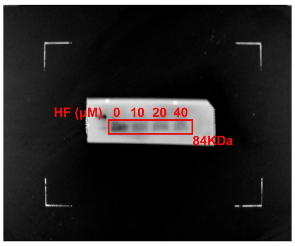

β-actin

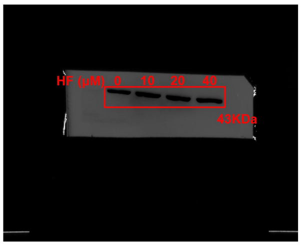

Protein quantification			
	MMP2	MMP9	β-actin
HF 0μM	34825.874	38877.095	38900.844
HF 10μM	31248.995	36122.016	38135.066
HF 20μM	20427.309	18873.894	39140.066
HF 40μM	8343.388	8568.439	40216.673

Relative expression		
	MMP2	MMP9
HF 0μM	1	1
HF 10μM	0.897292484	0.92913362
HF 20μM	0.586555531	0.485475934
HF 40μM	0.239574404	0.220398129

Figure 7B, C, E

For control group

0 day after treatment						
	Mouse No.1	Mouse No.2	Mouse No.3	Mouse No.4	Mouse No.5	Mouse No.6
Tumor (L)/mm	5.7	5.8	5.3	5.4	5.5	5.2
Tumor (W)/mm	4.5	4.1	4.2	4.4	4.3	4.3
Tumor (V)/mm^3	57.7125	48.749	46.746	52.272	50.8475	48.074
Body weight/g	16.2	15.3	16.3	16.4	16	16.1

3 days after treatment						
	Mouse No.1	Mouse No.2	Mouse No.3	Mouse No.4	Mouse No.5	Mouse No.6
Tumor (L)/mm	7.5	7.1	6.9	7.2	6.4	6.7
Tumor (W)/mm	5.6	5.9	6.2	5.7	6.3	6.2
Tumor (V)/mm^3	117.6	123.5755	132.618	116.964	127.008	128.774
Body weight/g	16.6	15.5	16.8	16.7	16.5	16.3

6 days after treatment						
	Mouse No.1	Mouse No.2	Mouse No.3	Mouse No.4	Mouse No.5	Mouse No.6
Tumor (L)/mm	12.1	10.1	11.3	11.5	9.5	10.5
Tumor (W)/mm	7	7.4	7.2	7.1	8	7.6
Tumor (V)/mm^3	296.45	276.538	292.896	289.8575	304	303.24
Body weight/g	16.9	16	17.1	16.9	17.2	17.1

9 days after treatment						
	Mouse No.1	Mouse No.2	Mouse No.3	Mouse No.4	Mouse No.5	Mouse No.6
Tumor (L)/mm	13.1	12	12.1	11.3	10	11.4
Tumor (W)/mm	8.4	8.6	8	8.7	9.1	9
Tumor (V)/mm^3	462.168	443.76	387.2	427.6485	414.05	461.7
Body weight/g	17.4	16.3	17.4	17.3	17.3	17.2

12 days after treatment						
	Mouse No.1	Mouse No.2	Mouse No.3	Mouse No.4	Mouse No.5	Mouse No.6
Tumor (L)/mm	13.4	11	12	12.4	11.5	11.5
Tumor (W)/mm	8.7	9.5	8.1	9	9.6	9.7
Tumor (V)/mm^3	507.123	496.375	393.66	502.2	529.92	541.0175
Body weight/g	18	17.2	18.3	18.1	18.4	18

15 days after treatment						
	Mouse No.1	Mouse No.2	Mouse No.3	Mouse No.4	Mouse No.5	Mouse No.6
Tumor (L)/mm	13.4	11	12.1	12.1	12	12
Tumor (W)/mm	8.8	9.6	8.2	9.5	9.7	9.8
Tumor (V)/mm^3	518.848	506.88	406.802	546.0125	564.54	576.24
Body weight/g	18.6	17.9	18.8	18.7	18.9	19

18 days after treatment						
	Mouse No.1	Mouse No.2	Mouse No.3	Mouse No.4	Mouse No.5	Mouse No.6
Tumor (L)/mm	13.3	11.2	12.8	12.3	12.2	12.1
Tumor (W)/mm	9	9.7	8.2	9.8	9.9	9.8
Tumor (V)/mm^3	538.65	526.904	430.336	590.646	597.861	581.042
Body weight/g	19.2	18.4	19.3	19.2	19.3	19.4

21 days after treatment						
	Mouse No.1	Mouse No.2	Mouse No.3	Mouse No.4	Mouse No.5	Mouse No.6
Tumor (L)/mm	13.8	13.2	13.1	13.6	12.3	13.1
Tumor (W)/mm	9.6	9.8	9	10	10.6	10.2
Tumor (V)/mm^3	635.904	633.864	530.55	680	691.014	681.462
Body weight/g	19.8	18.8	19.9	19.8	20.1	20

24 days after treatment						
	Mouse No.1	Mouse No.2	Mouse No.3	Mouse No.4	Mouse No.5	Mouse No.6
Tumor (L)/mm	14.7	13.6	14	13.8	12.7	13.5
Tumor (W)/mm	10.1	10.3	9.5	10.4	11.2	10.8
Tumor (V)/mm^3	749.7735	721.412	631.75	746.304	796.544	787.32
Body weight/g	20.8	19.9	21	21.3	21.2	21.4

For 25 mg/kg treatment group

0 day after treatment						
	Mouse No.1	Mouse No.2	Mouse No.3	Mouse No.4	Mouse No.5	Mouse No.6
Tumor (L)/mm	5.5	5.6	6	5.3	5	6.1
Tumor (W)/mm	4.3	4.1	4	4.5	4.5	4
Tumor (V)/mm^3	50.8475	47.068	48	53.6625	50.625	48.8
Body weight/g	16.5	16.6	16.9	16.3	16.2	16.8

3 days after treatment						
	Mouse No.1	Mouse No.2	Mouse No.3	Mouse No.4	Mouse No.5	Mouse No.6
Tumor (L)/mm	6.9	6.6	6.7	6.9	6.8	7.5
Tumor (W)/mm	5.6	5.5	5.7	5.4	5.5	5.3
Tumor (V)/mm^3	108.192	99.825	108.8415	100.602	102.85	105.3375
Body weight/g	16.9	17	17.1	16.8	16.9	17.3

6 days after treatment						
	Mouse No.1	Mouse No.2	Mouse No.3	Mouse No.4	Mouse No.5	Mouse No.6
Tumor (L)/mm	8.5	8.9	8.7	8.1	8.8	9.7
Tumor (W)/mm	7.4	7.2	7.1	7.4	7	6.8
Tumor (V)/mm^3	232.73	230.688	219.2835	221.778	215.6	224.264
Body weight/g	17.7	17.6	17.5	17.9	17.3	17.8

9 days after treatment						
	Mouse No.1	Mouse No.2	Mouse No.3	Mouse No.4	Mouse No.5	Mouse No.6
Tumor (L)/mm	9.1	11.6	9.2	8.4	9.4	10.1
Tumor (W)/mm	7.7	8.6	7.7	8.5	7.5	7.3
Tumor (V)/mm^3	269.7695	428.968	272.734	303.45	264.375	269.1145
Body weight/g	17.8	17.9	18.1	18.3	17.9	18

12 days after treatment						
	Mouse No.1	Mouse No.2	Mouse No.3	Mouse No.4	Mouse No.5	Mouse No.6
Tumor (L)/mm	9.2	11.8	9.4	8.5	9.6	10.2
Tumor (W)/mm	7.8	8.7	7.8	8.6	7.7	7.5
Tumor (V)/mm^3	279.864	446.571	285.948	314.33	284.592	286.875
Body weight/g	18.2	18.4	18.8	18.9	18	18.5

15 days after treatment						
	Mouse No.1	Mouse No.2	Mouse No.3	Mouse No.4	Mouse No.5	Mouse No.6
Tumor (L)/mm	9.3	12	9.5	8.7	9.7	10.3
Tumor (W)/mm	7.9	8.8	7.9	8.4	7.8	7.6
Tumor (V)/mm^3	290.2065	464.64	296.4475	306.936	295.074	297.464
Body weight/g	18.9	18.8	19.3	19.6	19.2	19.3

18 days after treatment						
	Mouse No.1	Mouse No.2	Mouse No.3	Mouse No.4	Mouse No.5	Mouse No.6
Tumor (L)/mm	9.3	12.1	9.6	8.9	9.7	10.5
Tumor (W)/mm	8	8.9	7.9	8.4	7.9	7.6
Tumor (V)/mm^3	297.6	479.2205	299.568	313.992	302.6885	303.24
Body weight/g	19.4	19.5	19.6	19.7	19.9	19.7

21 days after treatment						
	Mouse No.1	Mouse No.2	Mouse No.3	Mouse No.4	Mouse No.5	Mouse No.6
Tumor (L)/mm	9.3	12.2	9.6	9	9.7	10.6
Tumor (W)/mm	8.1	9	8	8.4	8	7.7
Tumor (V)/mm^3	305.0865	494.1	307.2	317.52	310.4	314.237
Body weight/g	20.4	20	20.5	20.3	20.4	20.7

24 days after treatment						
	Mouse No.1	Mouse No.2	Mouse No.3	Mouse No.4	Mouse No.5	Mouse No.6
Tumor (L)/mm	9.3	12.4	9.7	9.1	9.8	10.7
Tumor (W)/mm	8.2	9	8.1	8.4	8	7.8
Tumor (V)/mm^3	312.666	502.2	318.2085	321.048	313.6	325.494
Body weight/g	17.8	17.9	18.1	18.3	17.9	18

For 50 mg/kg treatment group

0 day after treatment						
	Mouse No.1	Mouse No.2	Mouse No.3	Mouse No.4	Mouse No.5	Mouse No.6
Tumor (L)/mm	6	5.2	5.7	6.2	5.5	5.4
Tumor (W)/mm	4	4.4	4.3	4.2	4.1	4.3
Tumor (V)/mm^3	48	50.336	52.6965	54.684	46.2275	49.923
Body weight/g	16.8	16.3	16.4	16.6	16.9	16.9

3 days after treatment						
	Mouse No.1	Mouse No.2	Mouse No.3	Mouse No.4	Mouse No.5	Mouse No.6
Tumor (L)/mm	6.2	5.7	6.2	6.6	6	5.8
Tumor (W)/mm	4.9	5	4.6	4.5	4.8	4.9
Tumor (V)/mm^3	74.431	71.25	65.596	66.825	69.12	69.629
Body weight/g	17.3	17	17	17.2	17.4	17.2

6 days after treatment						
	Mouse No.1	Mouse No.2	Mouse No.3	Mouse No.4	Mouse No.5	Mouse No.6
Tumor (L)/mm	6.4	5.9	6.3	6.8	6.1	6
Tumor (W)/mm	5.1	5.2	4.9	4.7	4.9	5
Tumor (V)/mm^3	83.232	79.768	75.6315	75.106	73.2305	75
Body weight/g	17.8	17.3	17.6	17.5	17.8	17.8

9 days after treatment						
	Mouse No.1	Mouse No.2	Mouse No.3	Mouse No.4	Mouse No.5	Mouse No.6
Tumor (L)/mm	6.5	6	6.4	6.9	6.2	6.1
Tumor (W)/mm	5.1	5.3	5	4.7	5	5.1
Tumor (V)/mm^3	84.5325	84.27	80	76.2105	77.5	79.3305
Body weight/g	18.3	18.4	18	18.1	18.5	18.1

12 days after treatment						
	Mouse No.1	Mouse No.2	Mouse No.3	Mouse No.4	Mouse No.5	Mouse No.6
Tumor (L)/mm	6.6	6	6.6	7.1	6.2	6.1
Tumor (W)/mm	5.3	5.4	5	5	5.1	5.3
Tumor (V)/mm^3	92.697	87.48	82.5	88.75	80.631	85.6745
Body weight/g	18.8	18.6	18.9	18.7	19	19.1

15 days after treatment						
	Mouse No.1	Mouse No.2	Mouse No.3	Mouse No.4	Mouse No.5	Mouse No.6
Tumor (L)/mm	6.9	6.2	6.7	7.3	6.4	6.3
Tumor (W)/mm	5.4	5.6	5.3	5.1	5.2	5.4
Tumor (V)/mm^3	100.602	97.216	94.1015	94.9365	86.528	91.854
Body weight/g	19.2	19.3	19	19.3	19.2	19.3

18 days after treatment						
	Mouse No.1	Mouse No.2	Mouse No.3	Mouse No.4	Mouse No.5	Mouse No.6
Tumor (L)/mm	7.1	6.4	6.8	7.5	6.5	6.8
Tumor (W)/mm	5.5	5.8	5.5	5.3	5.2	6.8
Tumor (V)/mm^3	107.3875	107.648	102.85	105.3375	87.88	157.216
Body weight/g	19.9	19.7	19.8	19.7	20	20.1

21 days after treatment						
	Mouse No.1	Mouse No.2	Mouse No.3	Mouse No.4	Mouse No.5	Mouse No.6
Tumor (L)/mm	7.3	6.6	7	7.8	6.5	6.8
Tumor (W)/mm	5.7	6	5.6	5.5	5.3	7
Tumor (V)/mm^3	118.5885	118.8	109.76	117.975	91.2925	166.6
Body weight/g	20.4	20.7	20	20.2	20.5	20.3

24 days after treatment						
	Mouse No.1	Mouse No.2	Mouse No.3	Mouse No.4	Mouse No.5	Mouse No.6
Tumor (L)/mm	7.4	6.7	7.2	8	6.6	6.9
Tumor (W)/mm	5.9	6.1	5.7	5.6	5.4	7
Tumor (V)/mm^3	128.797	124.6535	116.964	125.44	96.228	169.05
Body weight/g	20.9	21	20.8	20.7	21	20.9

Figure 7D

Tumor weight/(g)			
	Control	25 mg/kg	50 mg/kg
Mouse No.1	0.53	0.34	0.08
Mouse No.2	0.56	0.36	0.09
Mouse No.3	0.45	0.35	0.07
Mouse No.4	0.54	0.31	0.06
Mouse No.5	0.52	0.23	0.05
Mouse No.6	0.55	0.3	0.13

Figure 7F

p-AKT

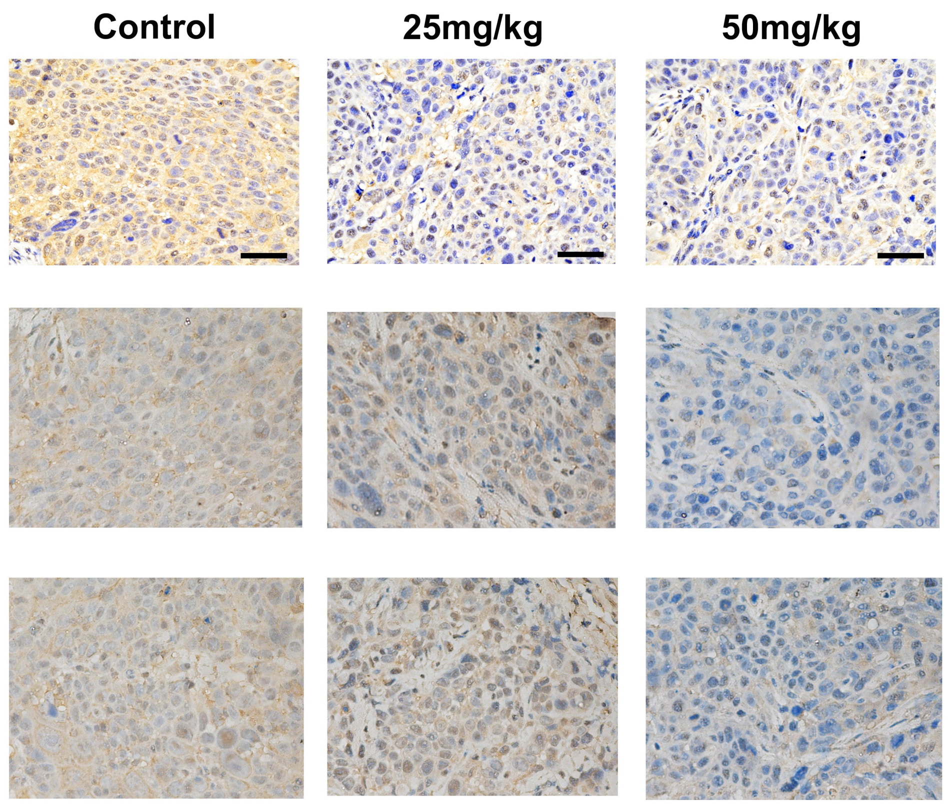

p-mTOR

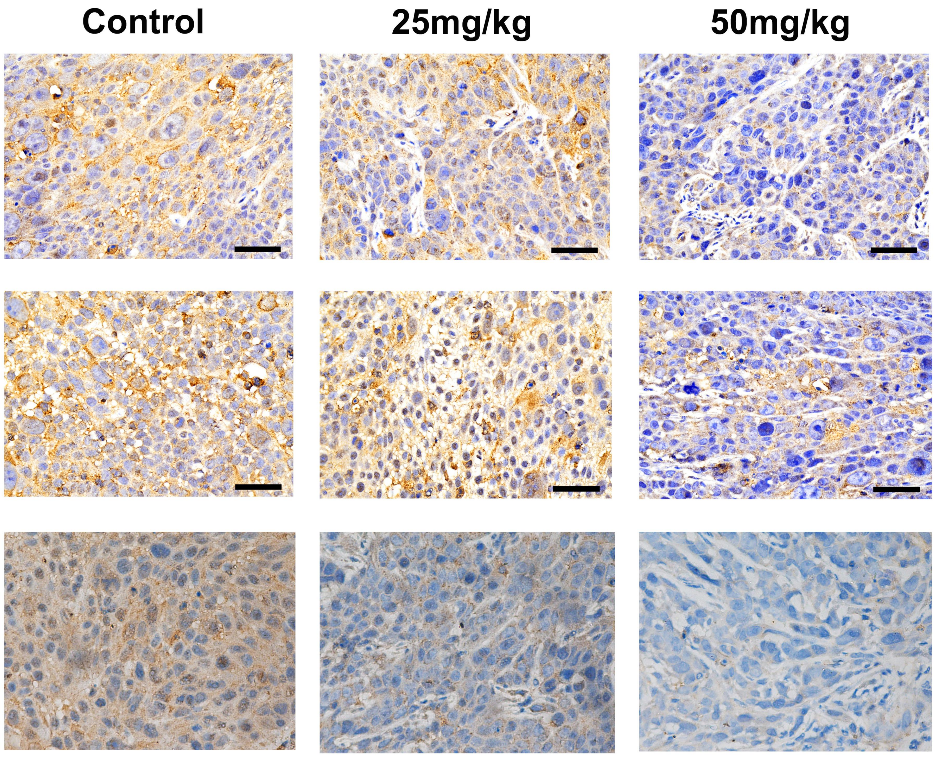

Bax

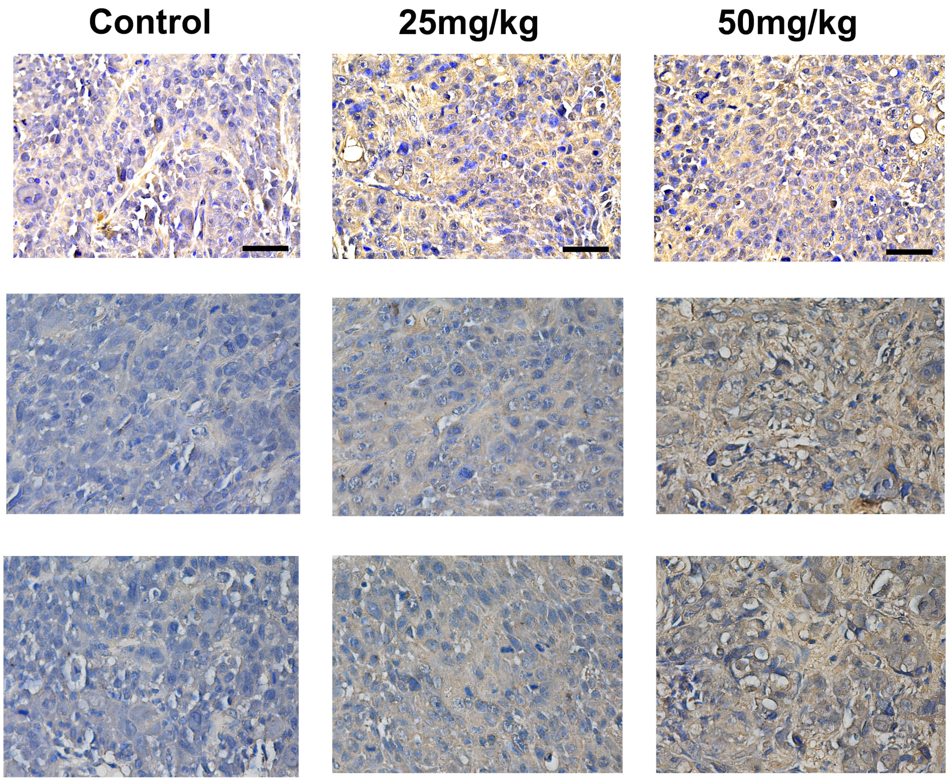

c-Caspase 3

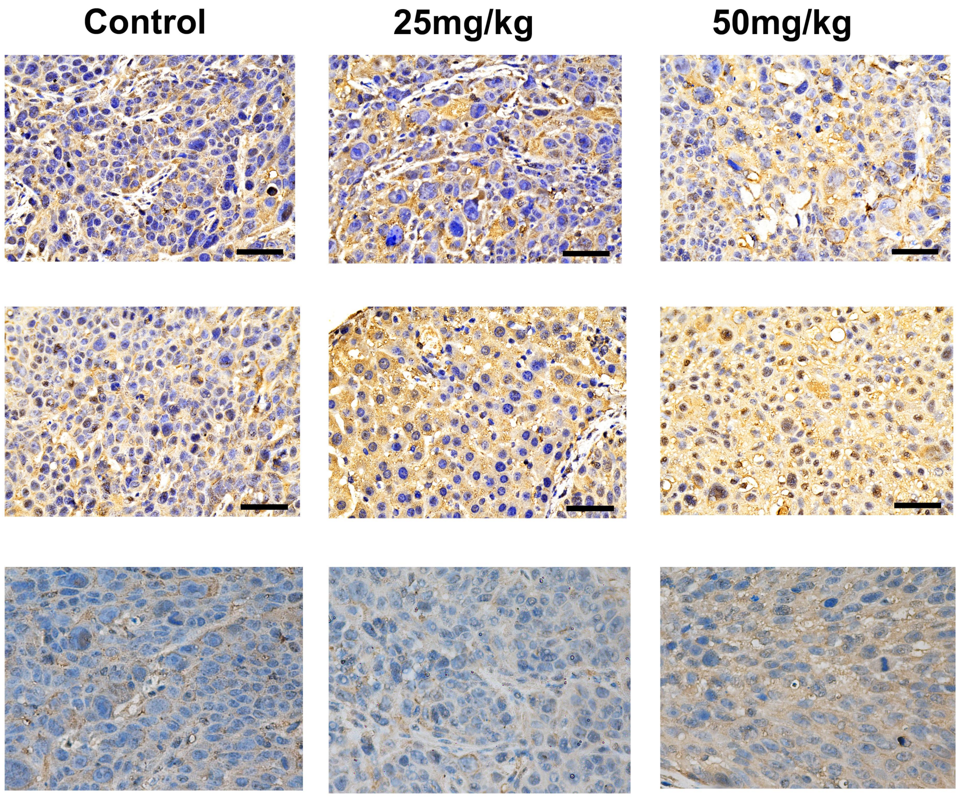

Figure 7G

Heart

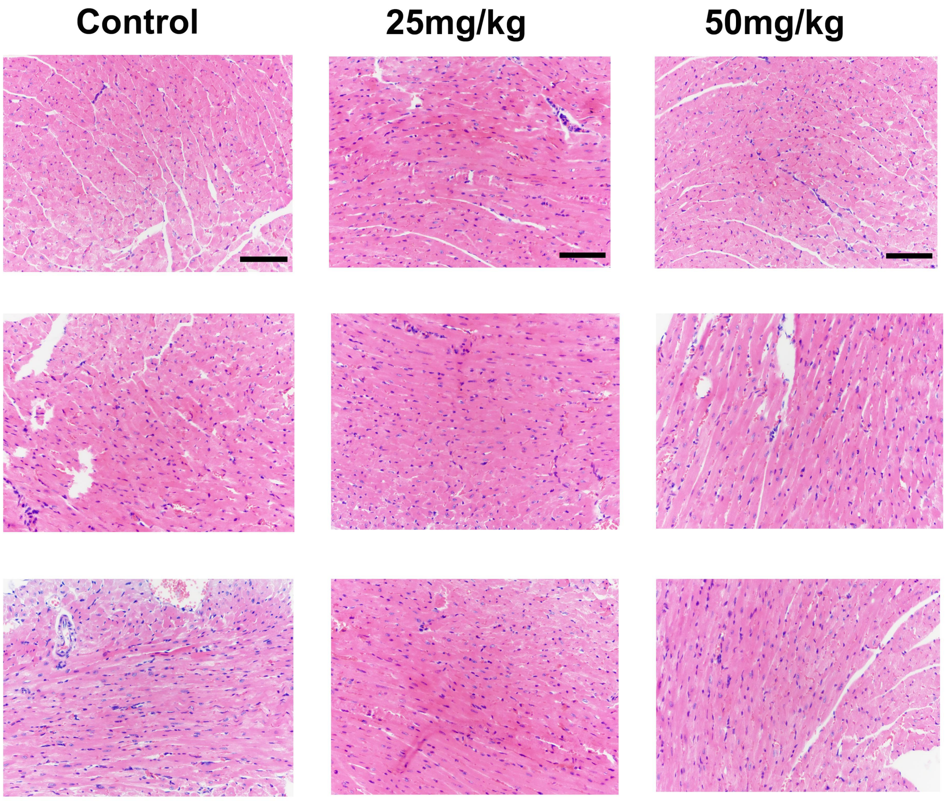

Liver

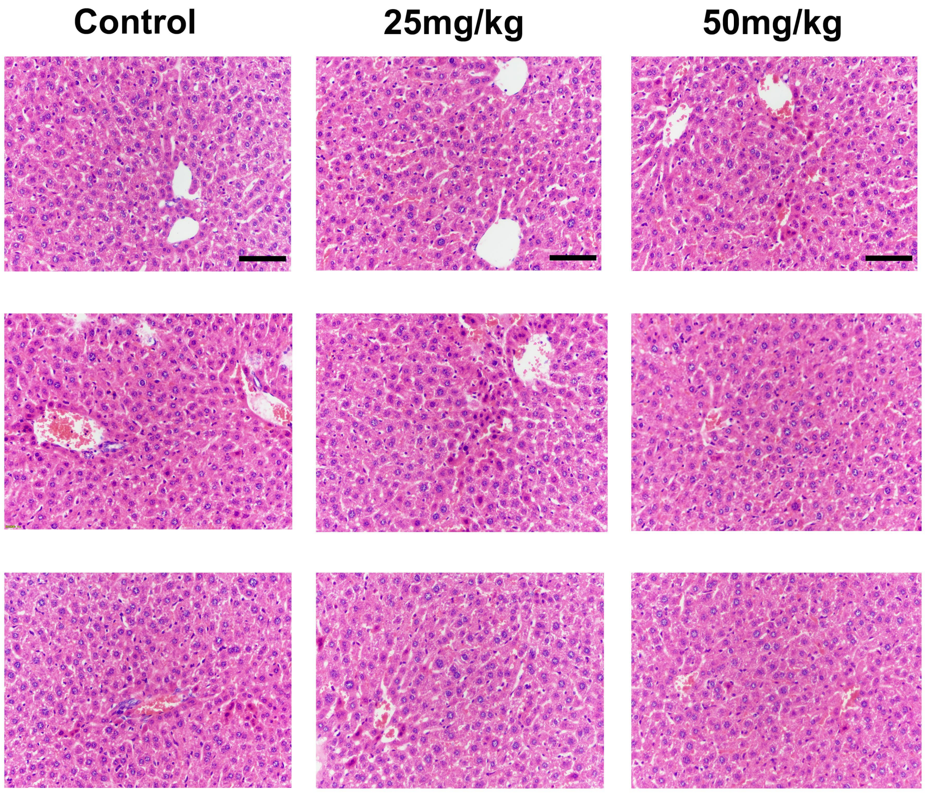

Spleen

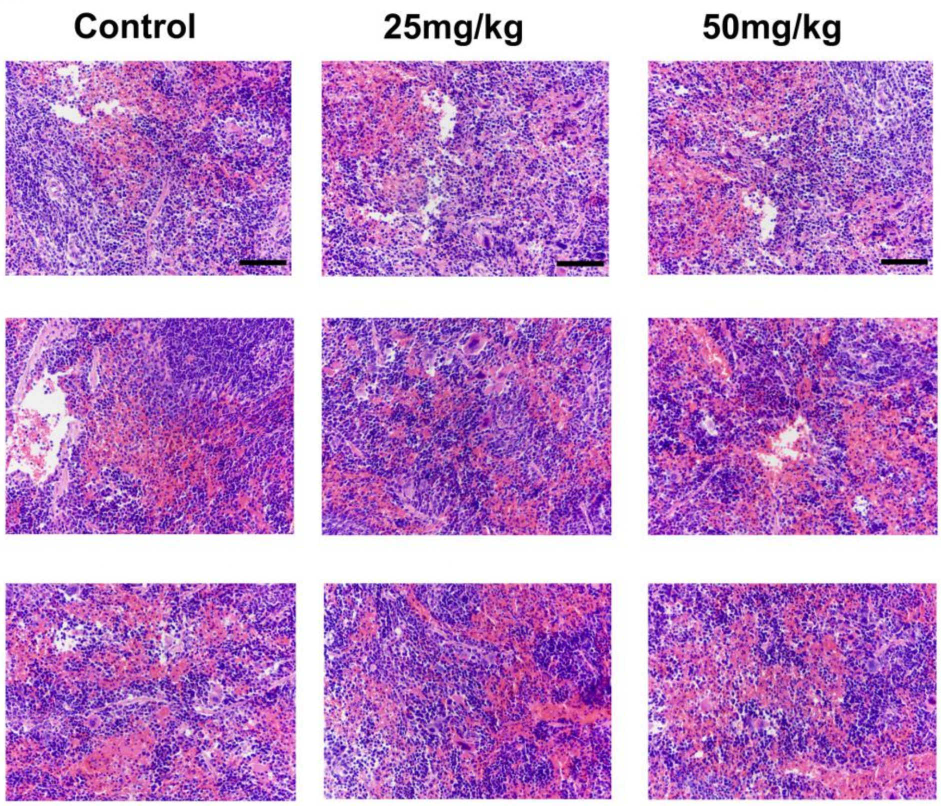

Lung

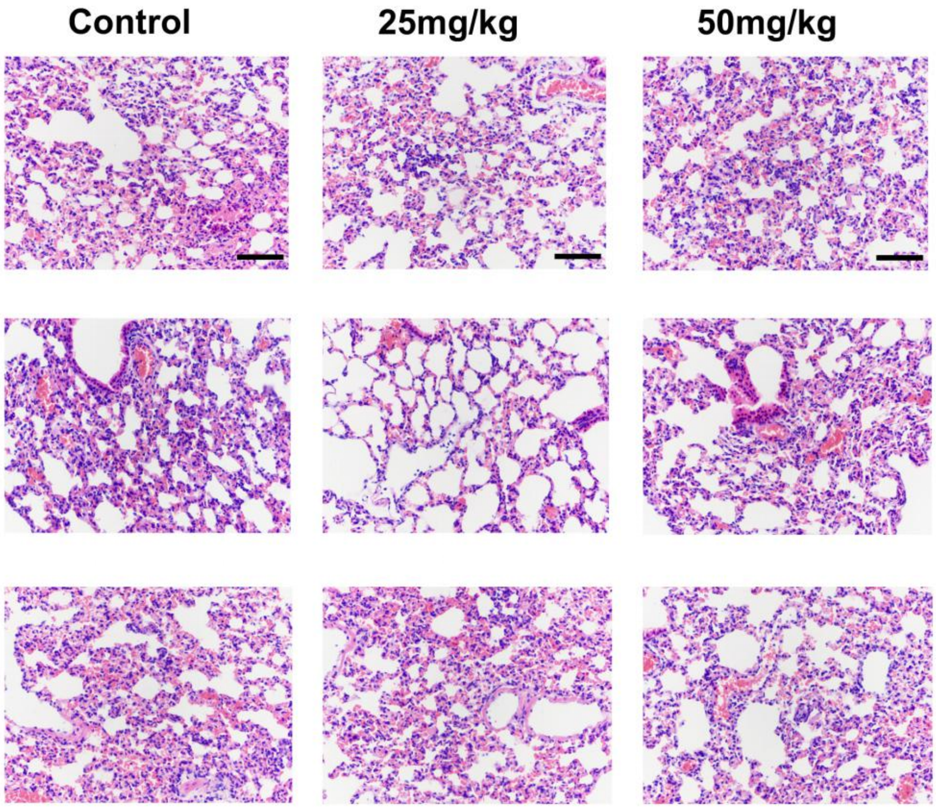

Kidney

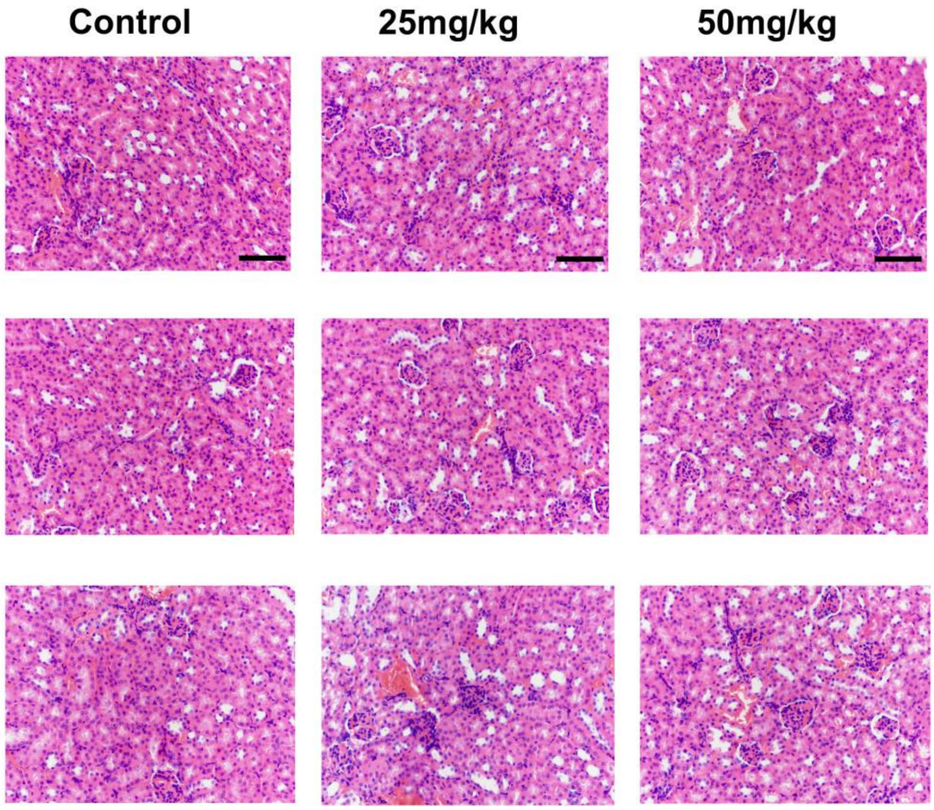

Supplement: Supplementary file 1 [file DataSheet_1.pdf]
