# Supplementary material for: Hinokiflavone Inhibits Growth of Esophageal Squamous Cancer By Inducing Apoptosis via Regulation of the PI3K/AKT/mTOR Signaling Pathway
Source: Front Oncol. 2022 Feb 1;12:833719. doi: 10.3389/fonc.2022.833719 (PMC8844566; doi:10.3389/fonc.2022.833719)
Supplement: Supplementary file 2 [file DataSheet_2.pdf]

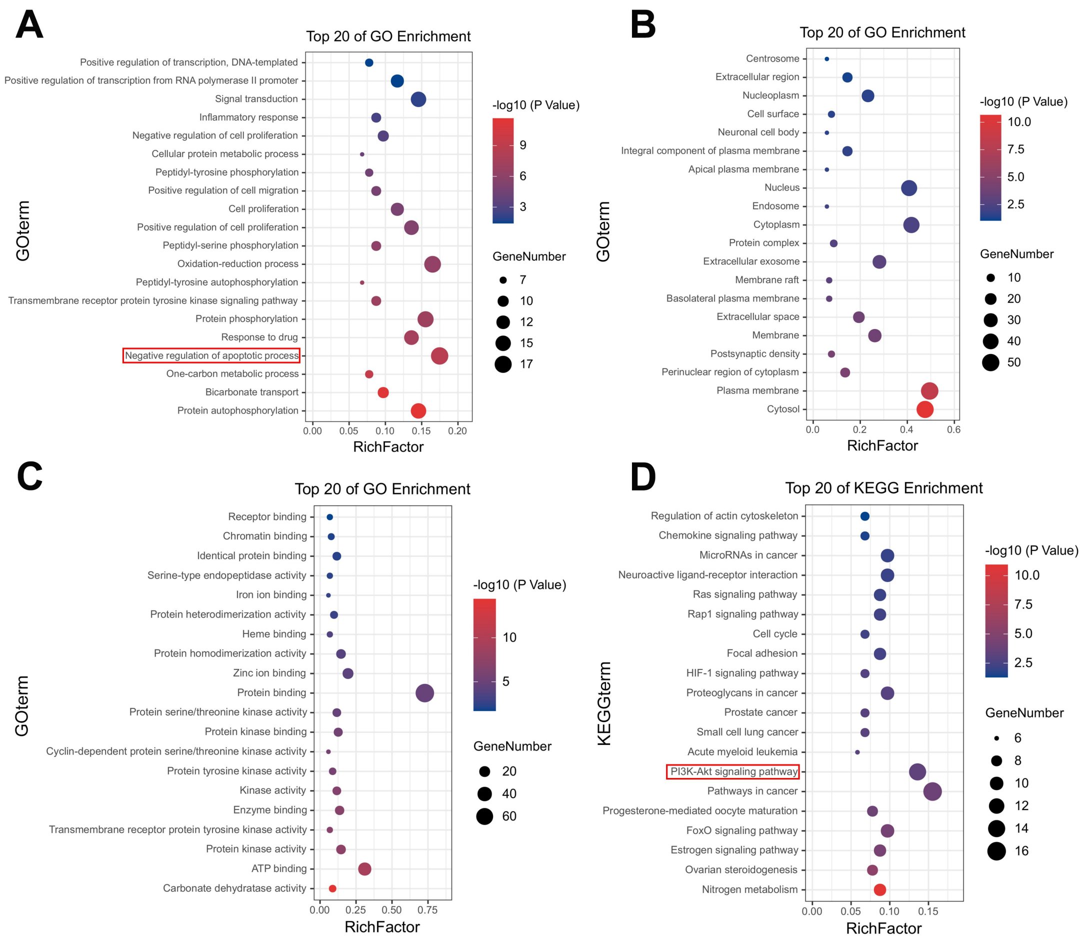

Figure S1: Gene Ontology (GO) and Kyoto Encyclopedia of Genes and Genomes (KEGG) pathway analysis of HF based on Swiss database. Top 20 signaling pathways of HF in **(A)** GO-BP pathway analysis, **(B)** GO-CC pathway analysis, and **(C)** GO-MF pathway analysis. **(D)** Top 20 signaling pathways of HF in KEGG enrichment pathway analysis.

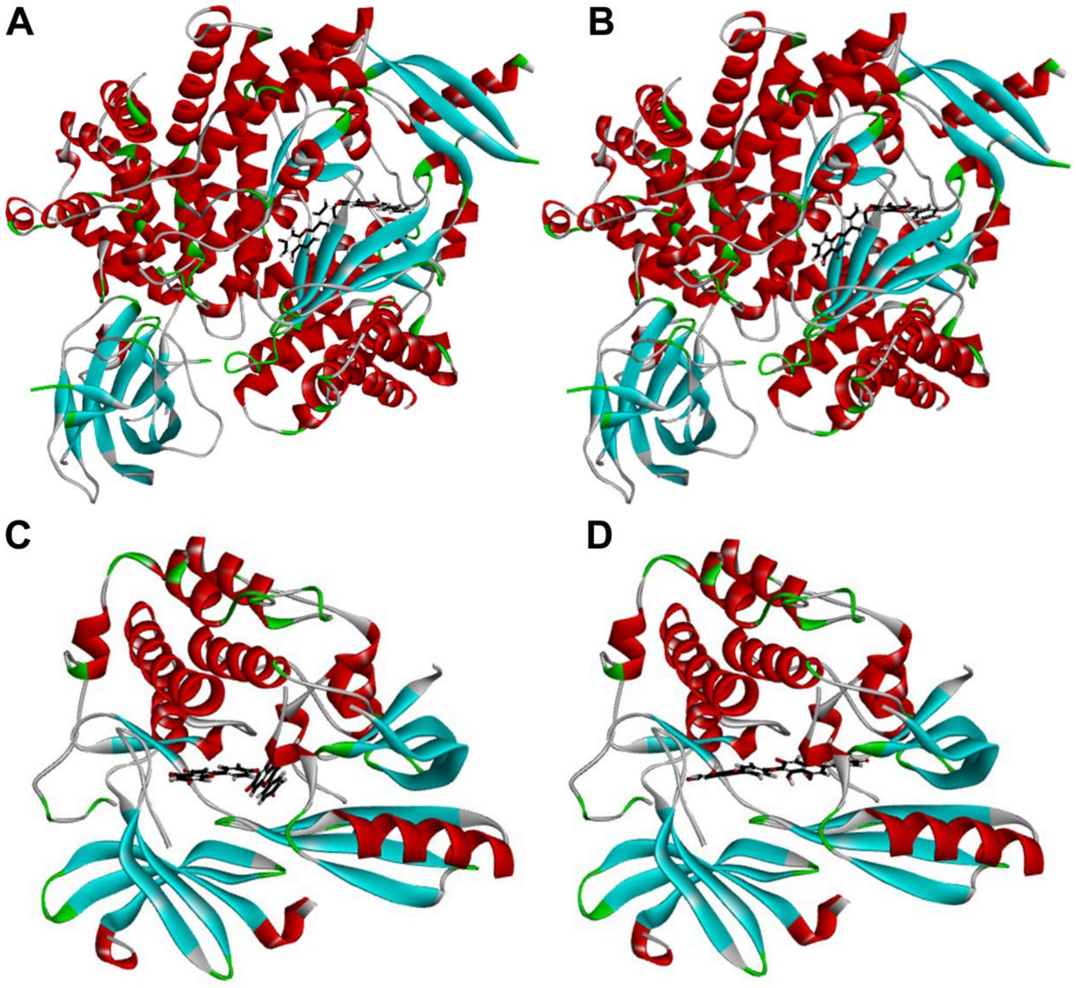

Figure S2: Three dimensional schematic of the molecular docking of other conformations of HF onto PI3K and AKT1. **(A)**, **(B)** Three-dimensional view of HF and PI3K docking scenario in two different conformations (LibDock score 121.363 and 118.8). **(C)**, **(D)** Three-dimensional view of HF and AKT1 docking scenario in two different conformations (LibDock score 156.149 and 144.279).

Table S1: The top 20 of the gene ontology (GO) function enrichment analysis of potential targets from Pharma Mapper database

Category	Term	Rich Factor %	P Value	Count
GO-BP	GO:0007165~signal transduction	15.40697674	3.78E-08	53
GO-BP	GO:0045944~positive regulation of transcription from RNA polymerase II promoter	12.20930233	6.79E-06	42
GO-BP	GO:0043066~negative regulation of apoptotic process	10.75581395	2.13E-12	37
GO-BP	GO:0055114~oxidation-reduction process	10.1744186	3.79E-08	35
GO-BP	GO:0006508~proteolysis	9.302325581	2.80E-08	32
GO-BP	GO:0006468~protein phosphorylation	8.430232558	1.65E-07	29
GO-BP	GO:0008284~positive regulation of cell proliferation	8.430232558	2.57E-07	29
GO-BP	GO:0006367~transcription initiation from RNA polymerase II promoter	8.139534884	3.17E-18	28
GO-BP	GO:0042493~response to drug	7.848837209	5.38E-10	27
GO-BP	GO:0046777~protein autophosphorylation	7.26744186	7.38E-14	25
GO-BP	GO:0045893~positive regulation of transcription, DNA-templated	7.26744186	1.21E-04	25
GO-BP	GO:0043401~steroid hormone mediated signaling pathway	6.686046512	4.09E-23	23
GO-BP	GO:0000122~negative regulation of transcription from RNA polymerase II promoter	6.686046512	0.033573836	23
GO-BP	GO:0008283~cell proliferation	6.395348837	1.69E-05	22
GO-BP	GO:0006915~apoptotic process	6.395348837	0.005349195	22
GO-BP	GO:0018108~peptidyl-tyrosine phosphorylation	6.104651163	3.16E-11	21
GO-BP	GO:0008285~negative regulation of cell proliferation	6.104651163	1.56E-04	21
GO-BP	GO:0035556~intracellular signal transduction	6.104651163	1.97E-04	21
GO-BP	GO:0001666~response to hypoxia	5.523255814	1.16E-08	19
GO-BP	GO:0045087~innate immune response	5.523255814	0.002769122	19
GO-CC	GO:0005829~cytosol	52.03488372	2.34E-47	179
GO-CC	GO:0005737~cytoplasm	43.60465116	8.03E-10	150
GO-CC	GO:0070062~extracellular exosome	40.40697674	4.46E-30	139
GO-CC	GO:0005634~nucleus	37.20930233	0.001059976	128
GO-CC	GO:0005886~plasma membrane	27.90697674	0.01006021	96
GO-CC	GO:0005654~nucleoplasm	27.61627907	1.95E-09	95
GO-CC	GO:0016020~membrane	19.76744186	2.63E-05	68
GO-CC	GO:0005615~extracellular space	18.89534884	2.20E-12	65
GO-CC	GO:0005576~extracellular region	18.89534884	3.79E-09	65
GO-CC	GO:0005739~mitochondrion	16.86046512	2.24E-09	58
GO-CC	GO:0048471~perinuclear region of	6.686046512	0.002983913	23

	cytoplasm			
GO-CC	GO:0005759~mitochondrial matrix	6.395348837	8.79E-07	22
GO-CC	GO:0009986~cell surface	6.395348837	0.001253836	22
GO-CC	GO:0005925~focal adhesion	5.523255814	3.95E-04	19
GO-CC	GO:0031012~extracellular matrix	4.941860465	1.40E-04	17
GO-CC	GO:0045121~membrane raft	4.651162791	7.38E-06	16
GO-CC	GO:0043231~intracellular membrane-bounded organelle	4.651162791	0.097071595	16
GO-CC	GO:0043234~protein complex	4.360465116	0.022116571	15
GO-CC	GO:0005764~lysosome	4.069767442	3.21E-04	14
GO-CC	GO:0043025~neuronal cell body	4.069767442	0.006241599	14
GO-MT	GO:0005515~protein binding	62.5	2.07E-05	215
GO-MT	GO:0005524~ATP binding	21.80232558	1.84E-13	75
GO-MT	GO:0008270~zinc ion binding	15.98837209	7.07E-09	55
GO-MT	GO:0042802~identical protein binding	13.6627907	1.26E-11	47
GO-MT	GO:0042803~protein homodimerization activity	11.91860465	8.30E-09	41
GO-MT	GO:0019899~enzyme binding	9.302325581	1.21E-12	32
GO-MT	GO:0004672~protein kinase activity	9.011627907	4.31E-11	31
GO-MT	GO:0003700~transcription factor activity, sequence-specific DNA binding	7.848837209	0.076038974	27
GO-MT	GO:0003707~steroid hormone receptor activity	7.558139535	4.75E-28	26
GO-MT	GO:0043565~sequence-specific DNA binding	7.558139535	5.09E-05	26
GO-MT	GO:0004674~protein serine/threonine kinase activity	7.26744186	6.63E-07	25
GO-MT	GO:0004713~protein tyrosine kinase activity	6.686046512	2.31E-14	23
GO-MT	GO:0016301~kinase activity	6.686046512	3.63E-09	23
GO-MT	GO:0005102~receptor binding	6.686046512	2.88E-06	23
GO-MT	GO:0019901~protein kinase binding	6.686046512	7.95E-06	23
GO-MT	GO:0004252~serine-type endopeptidase activity	6.395348837	5.03E-08	22
GO-MT	GO:0008144~drug binding	5.523255814	7.82E-15	19
GO-MT	GO:0003824~catalytic activity	5.523255814	4.73E-08	19
GO-MT	GO:0016491~oxidoreductase activity	4.941860465	2.94E-06	17
GO-MT	GO:0005525~GTP binding	4.941860465	0.004916445	17

Table S2: The top 20 of KEGG Pathway Enrichment Analyses of potential targets from Pharma Mapper database

Category	Term	Rich Factor %	P Value	Count
KEGG-PATHWAY	hsa01100:Metabolic pathways	27.03488372	6.85E-10	93
KEGG-PATHWAY	hsa05200:Pathways in cancer	13.6627907	6.10E-11	47
KEGG-PATHWAY	hsa04151:PI3K-Akt signaling pathway	10.75581395	1.86E-07	37
KEGG-PATHWAY	hsa01130:Biosynthesis of antibiotics	9.011627907	2.13E-09	31
KEGG-PATHWAY	hsa04014:Ras signaling pathway	8.720930233	3.82E-08	30
KEGG-PATHWAY	hsa05205:Proteoglycans in cancer	8.139534884	3.97E-08	28
KEGG-PATHWAY	hsa04015:Rap1 signaling pathway	6.976744186	1.59E-05	24
KEGG-PATHWAY	hsa04068:FoxO signaling pathway	6.686046512	2.17E-08	23
KEGG-PATHWAY	hsa04910:Insulin signaling pathway	6.686046512	3.77E-08	23
KEGG-PATHWAY	hsa04510:Focal adhesion	6.395348837	1.08E-04	22
KEGG-PATHWAY	hsa00230:Purine metabolism	6.104651163	3.39E-05	21
KEGG-PATHWAY	hsa05152:Tuberculosis	6.104651163	3.68E-05	21
KEGG-PATHWAY	hsa04010:MAPK signaling pathway	5.813953488	0.008210654	20
KEGG-PATHWAY	hsa05215:Prostate cancer	5.523255814	1.26E-08	19
KEGG-PATHWAY	hsa05161:Hepatitis B	5.523255814	2.57E-05	19
KEGG-PATHWAY	hsa04915:Estrogen signaling pathway	5.23255814	4.71E-07	18
KEGG-PATHWAY	hsa04931:Insulin resistance	5.23255814	1.68E-06	18
KEGG-PATHWAY	hsa04919:Thyroid hormone signaling pathway	5.23255814	4.09E-06	18
KEGG-PATHWAY	hsa04722:Neurotrophin signaling pathway	5.23255814	7.39E-06	18
KEGG-PATHWAY	hsa05203:Viral carcinogenesis	5.23255814	0.004633605	18

Table S3: The top 20 of the gene ontology (GO) function enrichment analysis of potential targets from Swiss database

Category	Term	Rich Factor %	P Value	Count
GO-BP	GO:0043066~negative regulation of apoptotic process	17.47572816	1.96E-09	18
GO-BP	GO:0055114~oxidation-reduction process	16.50485437	5.31E-07	17
GO-BP	GO:0006468~protein phosphorylation	15.53398058	1.03E-07	16
GO-BP	GO:0046777~protein autophosphorylation	14.5631068	2.23E-12	15
GO-BP	GO:0007165~signal transduction	14.5631068	0.010386567	15
GO-BP	GO:0042493~response to drug	13.59223301	3.84E-08	14
GO-BP	GO:0008284~positive regulation of cell proliferation	13.59223301	4.84E-06	14
GO-BP	GO:0008283~cell proliferation	11.65048544	1.37E-05	12
GO-BP	GO:0045944~positive regulation of transcription from RNA polymerase II promoter	11.65048544	0.03569245	12
GO-BP	GO:0015701~bicarbonate transport	9.708737864	4.67E-12	10
GO-BP	GO:0008285~negative regulation of cell proliferation	9.708737864	6.83E-04	10
GO-BP	GO:0007169~transmembrane receptor protein tyrosine kinase signaling pathway	8.737864078	1.20E-07	9
GO-BP	GO:0018105~peptidyl-serine phosphorylation	8.737864078	9.19E-07	9
GO-BP	GO:0030335~positive regulation of cell migration	8.737864078	1.63E-05	9
GO-BP	GO:0006954~inflammatory response	8.737864078	0.002168912	9
GO-BP	GO:0006730~one-carbon metabolic process	7.766990291	4.50E-10	8
GO-BP	GO:0018108~peptidyl-tyrosine phosphorylation	7.766990291	4.07E-05	8
GO-BP	GO:0045893~positive regulation of transcription, DNA-templated	7.766990291	0.037381048	8
GO-BP	GO:0038083~peptidyl-tyrosine autophosphorylation	6.796116505	1.41E-07	7
GO-BP	GO:0044267~cellular protein metabolic process	6.796116505	8.24E-05	7
GO-CC	GO:0005886~plasma membrane	49.51456311	4.20E-09	51
GO-CC	GO:0005829~cytosol	47.57281553	2.19E-11	49
GO-CC	GO:0005737~cytoplasm	41.74757282	0.004411007	43
GO-CC	GO:0005634~nucleus	40.77669903	0.015066776	42
GO-CC	GO:0070062~extracellular exosome	28.15533981	0.001290672	29
GO-CC	GO:0016020~membrane	26.21359223	1.47E-04	27
GO-CC	GO:0005654~nucleoplasm	23.30097087	0.032765441	24
GO-CC	GO:0005615~extracellular space	19.41747573	1.54E-04	20
GO-CC	GO:0005887~integral component of plasma membrane	14.5631068	0.025949188	15
GO-CC	GO:0005576~extracellular region	14.5631068	0.064629709	15
GO-CC	GO:0048471~perinuclear region of cytoplasm	13.59223301	4.26E-05	14

GO-CC	GO:0043234~protein complex	8.737864078	0.002197903	9
GO-CC	GO:0014069~postsynaptic density	7.766990291	7.86E-05	8
GO-CC	GO:0009986~cell surface	7.766990291	0.032498798	8
GO-CC	GO:0016323~basolateral plasma membrane	6.796116505	5.25E-04	7
GO-CC	GO:0045121~membrane raft	6.796116505	0.001061451	7
GO-CC	GO:0005768~endosome	5.825242718	0.008679528	6
GO-CC	GO:0016324~apical plasma membrane	5.825242718	0.023836082	6
GO-CC	GO:0043025~neuronal cell body	5.825242718	0.032029473	6
GO-CC	GO:0005813~centrosome	5.825242718	0.090964998	6
GO-MT	GO:0005515~protein binding	72.81553398	1.77E-05	75
GO-MT	GO:0005524~ATP binding	31.06796117	4.70E-10	32
GO-MT	GO:0008270~zinc ion binding	19.41747573	6.47E-05	20
GO-MT	GO:0004672~protein kinase activity	14.5631068	3.29E-08	15
GO-MT	GO:0042803~protein homodimerization activity	14.5631068	1.23E-04	15
GO-MT	GO:0019899~enzyme binding	13.59223301	1.05E-07	14
GO-MT	GO:0019901~protein kinase binding	12.62135922	2.79E-06	13
GO-MT	GO:0016301~kinase activity	11.65048544	2.27E-07	12
GO-MT	GO:0004674~protein serine/threonine kinase activity	11.65048544	1.68E-05	12
GO-MT	GO:0042802~identical protein binding	11.65048544	0.005527812	12
GO-MT	GO:0046982~protein heterodimerization activity	9.708737864	0.002012056	10
GO-MT	GO:0004089~carbonate dehydratase activity	8.737864078	3.90E-15	9
GO-MT	GO:0004713~protein tyrosine kinase activity	8.737864078	1.42E-06	9
GO-MT	GO:0003682~chromatin binding	7.766990291	0.009535505	8
GO-MT	GO:0004714~transmembrane receptor protein tyrosine kinase activity	6.796116505	9.90E-08	7
GO-MT	GO:0020037~heme binding	6.796116505	1.82E-04	7
GO-MT	GO:0004252~serine-type endopeptidase activity	6.796116505	0.004509241	7
GO-MT	GO:0005102~receptor binding	6.796116505	0.020182276	7
GO-MT	GO:0004693~cyclin-dependent protein serine/threonine kinase activity	5.825242718	1.77E-06	6
GO-MT	GO:0005506~iron ion binding	5.825242718	0.002356184	6

Table S4: The top 20 of KEGG Pathway Enrichment Analyses of potential targets from Swiss database

Category	Term	Rich Factor %	P Value	Count
KEGG-PATHWAY	hsa05200:Pathways in cancer	15.53398058	1.18E-04	16
KEGG-PATHWAY	hsa04151:PI3K-Akt signaling pathway	13.59223301	3.99E-04	14
KEGG-PATHWAY	hsa04068:FoxO signaling pathway	9.708737864	4.80E-05	10
KEGG-PATHWAY	hsa05205:Proteoglycans in cancer	9.708737864	9.72E-04	10
KEGG-PATHWAY	hsa04080:Neuroactive ligand-receptor interaction	9.708737864	0.008612731	10
KEGG-PATHWAY	hsa05206:MicroRNAs in cancer	9.708737864	0.010504379	10
KEGG-PATHWAY	hsa00910:Nitrogen metabolism	8.737864078	1.15E-11	9
KEGG-PATHWAY	hsa04915:Estrogen signaling pathway	8.737864078	3.45E-05	9
KEGG-PATHWAY	hsa04510:Focal adhesion	8.737864078	0.004656157	9
KEGG-PATHWAY	hsa04015:Rap1 signaling pathway	8.737864078	0.005224474	9
KEGG-PATHWAY	hsa04014:Ras signaling pathway	8.737864078	0.008046198	9
KEGG-PATHWAY	hsa04913:Ovarian steroidogenesis	7.766990291	2.45E-06	8
KEGG-PATHWAY	hsa04914:Progesterone-medi ated oocyte maturation	7.766990291	1.12E-04	8
KEGG-PATHWAY	hsa05222:Small cell lung cancer	6.796116505	7.19E-04	7
KEGG-PATHWAY	hsa05215:Prostate cancer	6.796116505	8.64E-04	7
KEGG-PATHWAY	hsa04066:HIF-1 signaling pathway	6.796116505	0.001362326	7
KEGG-PATHWAY	hsa04110:Cell cycle	6.796116505	0.004938346	7
KEGG-PATHWAY	hsa04062:Chemokine signaling pathway	6.796116505	0.03139023	7
KEGG-PATHWAY	hsa04810:Regulation of actin cytoskeleton	6.796116505	0.051641747	7
KEGG-PATHWAY	hsa05221:Acute myeloid leukemia	5.825242718	6.99E-04	6
