# Supplementary material for: Hinokiflavone Inhibits Growth of Esophageal Squamous Cancer By Inducing Apoptosis via Regulation of the PI3K/AKT/mTOR Signaling Pathway
Source: Front Oncol. 2022 Feb 1;12:833719. doi: 10.3389/fonc.2022.833719 (PMC8844566; doi:10.3389/fonc.2022.833719)
Supplement: Supplementary file 4 [file Table_2.docx]

Worksheets of publicly available datasets and raw data images download are presented below link: https://www.jianguoyun.com/p/DXzsjYcQ15SOChiU2qEE
